# Identification and experimental validation of CD74, PGLYRP1, and TXN as potential biomarkers in rheumatoid arthritis: an integrative bulk and ScRNA-seq study

**DOI:** 10.3389/fimmu.2026.1824952

**Published:** 2026-06-23

**Authors:** Yun Zheng, Tihui Wang, Jiyue Huang, Peng Niu, Xiu Yang, Jie Xiao, Guanyin Wu, Xiaotang Sun, Jinquan Li, Hui Wang

**Affiliations:** 1 Department of Orthopedic Surgery, 900th Hospital of PLA Joint Logistic Support Force, Fuzhou, Fujian, China; 2 Department of Orthopaedics, Mindong Hospital Affiliated to Fujian Medical University, Fuzhou, Fujian, China; 3 Department of Spine and Joint Surgery, Second General Hospital of Nanyang, Nanyang, Henan, China; 4 Fuzong Clinical Medical College of Fujian Medical University, Fuzhou, Fujian, China

**Keywords:** biomarkers, bulk, immunity, rheumatoid arthritis, single-cell sequencing analysis

## Abstract

Chronic joint inflammation, the hallmark of rheumatoid arthritis (RA), is an autoimmune condition that commonly leads to progressive joint damage and dysfunction. While several clinical biomarkers are available for diagnosing and predicting RA, their specificity and sensitivity are still insufficient. Therefore, the objective was to discover biomarkers associated with RA and delineate their functional mechanisms. Methods: Publicly available RA transcriptomic datasets were utilized in this study. A combination of machine learning algorithms and expression validation led to the identification of relevant biomarkers. To elucidate their functional roles in RA, we performed enrichment analysis, immune microenvironment profiling, computational screening of compound-protein binding affinities, molecular docking, and molecular dynamics simulations (MDs). In parallel, single-cell RNA sequencing (scRNA-seq) was employed to pinpoint critical cell subsets and track changes in biomarker expression. Finally, biomarker levels were validated in clinical samples using reverse transcription quantitative PCR (RT-qPCR), western blotting (WB), and immunohistochemical (IHC) staining. Results: CD74, PGLYRP1, and TXN were identified as potential biomarkers. Their enrichment in pathways associated with immune response, inflammation, and redox processes highlights their possible roles in RA. Additionally, CD56dim natural killer cells displayed a marked positive association with CD74 (cor = 0.66, P < 0.001) and the strongest negative association with TXN (cor = -0.75, P < 0.001). Bergamottin and diphenylcyclopropenone exhibited high binding affinities for CD74 and TXN, respectively. MDs simulations confirmed the stability of these complexes. In a pilot analysis, scRNA-seq indicated myeloid cells as the potential key cell population. During myeloid cell differentiation, CD74 and TXN expression levels initially increased and then declined. RT-qPCR, WB, and IHC analyses consistently demonstrated that CD74 expression was significantly downregulated, whereas PGLYRP1 and TXN were markedly upregulated in RA clinical samples compared with controls, confirming the reliability of the bioinformatics predictions and supporting their potential roles as RA biomarkers. Conclusion: By integrating bulk RNA sequencing with scRNA-seq, CD74, PGLYRP1, and TXN were identified as biomarkers, with myeloid cells suggested as a potential key cell type, providing new insights into RA diagnosis and meriting further investigation of their functional roles.

## 1 Introduction

As a chronic systemic autoimmune disease, rheumatoid arthritis (RA) features enduring synovial inflammation accompanied by joint pain, swelling, morning stiffness, and loss of function (1). The exact pathogenesis is not fully understood, but current evidence implicates a combination of genetic susceptibility and environmental factors (2). Globally, the disease presents a considerable epidemiological burden, with a significantly higher prevalence in women and typical onset during middle age (3). Currently, treatments for RA include pharmacotherapy such as non-steroidal anti-inflammatory drugs (NSAIDs), corticosteroids, and disease-modifying anti-rheumatic drugs (DMARDs), as well as biologic agents (e.g., anti-tumor necrosis factor (TNF) therapies) (4–6). These treatment approaches can alleviate inflammation and reduce symptoms to some extent, but they each have limitations. Traditional medications may cause adverse effects such as gastrointestinal irritation, immunosuppression, and liver or kidney damage. Although biologic agents have shown notable efficacy in clinical practice, their high cost, variability in individual response, and potential for resistance make treatment strategies complex. Therefore, while current therapies can control the disease to a certain degree, they do not cure RA and cannot completely avoid side effects during treatment.

The occurrence of RA is closely associated with specific proteins and their encoding genes, which play critical roles in immune responses and may also influence joint tissue damage and repair processes (7). In RA, immune hyperactivation is frequently associated with alterations in specific genes. The proteins they encode, including tumor necrosis factor-α (TNF-α) and interleukin-6 (IL-6), have been validated as key contributors to RA pathogenesis (8). As research into RA-related genes and their protein products advances, a growing number of candidate biomarkers have been identified for early diagnosis and disease prediction. For example, by measuring the levels of anti-citrullinated peptide antibodies (ACPA), it is possible to predict the development of RA before clinical symptoms appear (9), and detection of anti-cyclic citrullinated peptide (anti-CCP) antibodies can help assess disease activity (10). However, existing biomarkers remain limited in specificity and sensitivity, unable to fully meet the demands of precision medicine, highlighting the urgent need to identify more reliable molecular markers for RA. Therefore, in-depth exploration of the roles of RA-related genes and proteins can enhance our understanding of RA pathogenesis and drive the transition of RA management toward precision medicine.

Single-cell RNA sequencing (scRNA-seq) holds substantial importance in biomedical research by enabling transcriptome profiling at single-cell resolution, thereby uncovering cellular heterogeneity, rare subpopulations, and the intricate architecture of tissue microenvironments (11, 12). This technology is particularly useful for uncovering detailed disease mechanisms and provides potential new targets for precision therapy. By contrast, traditional bulk RNA sequencing cannot provide details at the individual cell level but does offer the average gene expression profile of an entire sample, with the advantages of large sample size, stable quantification, and lower noise (13). The integration of scRNA-seq with bulk RNA-seq allows complementary insights at different levels: the former identifies potential key cell populations, while the latter can verify the expression of relevant molecules in large cohorts and analyze their correlations with clinical features (14).

This study integrated bulk and scRNA-seq data to identify RA-related biomarkers and investigated their roles from multiple dimensions, including cell type specificity, immune microenvironment, and molecular regulation. Predictions from bioinformatic analyses were further validated in clinical samples at transcriptional, protein, and tissue levels to enhance reliability and translational relevance. These findings provide novel perspectives on RA pathogenesis and lay a theoretical groundwork for the development of accurate diagnostic markers and therapeutic targets.

## 2 Materials and methods

### 2.1 Data acquisition

This research employed three RA datasets downloaded from the Gene Expression Omnibus (GEO) database (https://www.ncbi.nlm.nih.gov/geo/): GSE15573 (platform: GPL6102), GSE17755 (platform: GPL1291), and GSE289019 (platform: GPL24676). GSE15573, containing peripheral blood mononuclear cell (PBMC) from 18 RA patients and 15 controls, was designated the training set. GSE17755, with peripheral blood cells from 112 RA patients and 45 controls, was used for validation. scRNA-seq dataset, GSE289019, contained PBMC samples from one RA patient and one healthy control. In addition, we selected peripheral blood mononuclear cell data from 18 RA patients and 18 healthy controls in the CZ CELLxGENE Discover database (https://cellxgene.cziscience.com/) for validation (15). Finally, this study utilized the PPA database (https://proteome-phenome-atlas.com/) to screen for protein-coding genes associated with RA. The PPA database serves as an open-access platform that integrates extensive information on plasma protein expression and disease phenotypes across large populations, offering association data for approximately 3,000 proteins with over 1,000 diseases and traits. A search was conducted with “Rheumatoid Arthritis” as the keyword, with a significance threshold set at 0.05 to screen for proteins significantly associated with RA. Subsequently, a search of the National Center for Biotechnology Information (NCBI) database (https://www.ncbi.nlm.nih.gov/gene/) based on the names of the screened proteins retrieved 1,506 associated protein-coding genes relevant to RA (**Supplementary Table 1**). All the aforementioned data were obtained on August 1, 2025.

### 2.2 Analysis of differential gene expression

Differentially expressed genes (DEGs) between RA and control samples in GSE15573 were screened using the limma package (v 3.58.1) (16) based on |log_2_ fold change (FC)| > 0.5 and adj.P < 0.05. A volcano plot generated with ggplot2 package (v 3.5.2) (17) highlighted the top 10 up- and down-regulated genes (ranked by log2FC). The ComplexHeatmap package (v 2.21.1) (18) was then used to create a heatmap showing the expression levels of these 20 genes.

### 2.3 Functional enrichment analysis and protein-protein interaction network construction of candidate genes

The ggvenn package (v 0.1.10) (19) was utilized to analyze the overlap between DEGs and 1,506 protein-coding genes linked to RA. These overlapping genes were identified as candidate genes. Subsequently, To explore functional annotations, the clusterProfiler package (v 4.8.3) (20) was applied for Gene Ontology (GO) and Kyoto Encyclopedia of Genes and Genomes (KEGG) enrichment analyses (P < 0.05), revealing the candidate genes’ involvement in specific molecular functions (MFs), cellular components (CCs), and biological processes (BPs), and signaling pathways. Additionally, PPI networks were constructed using interactions from the Search Tool for the Retrieval of Interacting Genes (STRING) database (https://www.string-db.org, confidence > 0.4) and visualized with the circlize package (v 0.4.16) (21). Subsequently, genes that exhibited protein-level interactions were defined as hub genes.

### 2.4 Identification of biomarkers using machine learning algorithms, expression validation, and receiver operating characteristic analysis

Hub genes in GSE15573 were analyzed using least absolute shrinkage and selection operator (LASSO) regression and support vector machine-recursive feature elimination (SVM-RFE) to pinpoint RA-related characteristic genes. LASSO regression with the glmnet package (v 4.1.8) (22) and five-fold cross-validation selected genes with non-zero coefficients (LASSO-characteristic genes) at the optimal lambda. Subsequently, SVM-RFE implemented in the caret package (v 6.0.94) (23) with five-fold cross-validation iteratively eliminated low-importance features to refine the gene set. SVM-RFE-characteristic genes were defined as those present at the model’s maximum accuracy. Subsequently, the ggvenn package (v 0.1.10) was used to obtain the intersection of LASSO- and SVM-RFE-selected genes. From these, genes exhibiting significant differential expression (P < 0.05) and uniform directional changes in both GSE15573 and GSE17755 (Wilcoxon test) were designated as candidate biomarkers. ROC curves generated with the pROC package (v 1.18.0) (24) were used to evaluate diagnostic value, and candidates with area under the curve (AUC) > 0.7 in both datasets were selected as final biomarkers.

### 2.5 Establishment and assessment of the nomogram

A nomogram was developed using the rms package (v 6.5.0) (25) to assess the predictive probability of RA onset based on biomarkers from GSE15573. A nomogram was constructed by assigning point values to each biomarker, where the total score correlated with RA risk. Calibration was assessed using the regplot package (v 1.1) (26) and the Hosmer-Lemeshow (HL) test (P > 0.05). The pROC package (v 1.18.0) was employed to generate an ROC curve, and an AUC > 0.7 confirmed the model’s strong diagnostic performance. Decision curve analysis (DCA) using the ggDCA package (v 1.2) (27) was additionally conducted to evaluate clinical applicability. Finally, in the validation set GSE17755, model performance was evaluated using bootstrap sampling, with AUC values and their confidence intervals reported, and calibration curves plotted to validate the predictive performance of the nomogram.

### 2.6 Subcellular localization and tissue/organ-biomarker expression

In subcellular localization analysis, this study aimed to determine the specific intracellular distribution of biomarkers, thereby inferring their potential biological functions and mechanisms of action. Subcellular localization of the biomarker proteins was predicted using the GeneCards database (https://www.genecards.org, accessed on August 12, 2025). Gene annotation analysis was further conducted to delineate their expression patterns and potential functional roles across diverse tissues and organs. Specifically, the BioGPS database (http://biogps.org, accessed on August 12, 2025) was utilized to annotate the tissue-level mRNA expression of biomarkers. Built upon microarray data, BioGPS encompasses gene expression profiles across a diverse range of human tissues and organs, and is frequently employed for exploring tissue-specific gene expression patterns. The analysis workflow comprised the following steps: Firstly, the biomarker was searched on the platform to obtain its mRNA expression data across various human tissues. Subsequently, the average expression level of the biomarker in each tissue was calculated and compared with the global average. Finally, the selection of tissues and organs with expression levels exceeding the global average was undertaken, indicating a notable expression preference in these particular tissues.

### 2.7 Gene set enrichment analysis

GSEA was employed to thoroughly investigate the relationships between biomarkers and signaling pathways, thereby uncovering their underlying molecular mechanisms. The specific procedure was implemented as follows: Initially, the psych package (v 2.4.3) (28) was utilized to calculate the Spearman correlation coefficients between each biomarker and all other genes. Subsequently, following the ranking of genes based on their coefficients, GSEA was carried out via the clusterProfiler package (v 4.8.3). The analysis relied on the “C2:CP: KEGG gene sets” obtained from the Molecular Signatures Database (MSigDB) (https://www.gsea-msigdb.org/), with enrichment significance defined by P < 0.05, false discovery rate (FDR) < 0.25, |normalized enrichment score (NES)| > 1, and considered only gene sets containing between 5 and 5,000 genes.

### 2.8 Immune microenvironment analysis

Immune cell infiltration in GSE15573 was assessed by applying the ssGSEA algorithm (28 immune cell types) via the GSVA package (v 1.50.0) (29). Wilcoxon testing revealed cell types with significant abundance differences between RA and control samples (P < 0.05). Spearman correlation analysis, conducted with the psych package (v 2.4.3), examined associations between biomarkers and these differentially infiltrated immune cells, as well as correlations among the immune cells themselves, using criteria of |cor| > 0.3 and P < 0.05.

### 2.9 Construction of molecular regulatory network

o investigate the microRNAs (miRNAs) and transcription factors (TFs) governing RA-associated biomarkers, a molecular regulatory network was established. Specifically, the ENCODE database was accessed via the miRnet platform (https://www.mirnet.ca/, accessed on 12 August 2025) to predict miRNAs associated with biomarkers. Subsequently, miRNAs targeting two or more biomarkers simultaneously were identified as key miRNAs. Following TF prediction for the biomarkers via the miRnet platform, a comprehensive miRNA-biomarker-TF regulatory network was established using Cytoscape software (v 3.10.1) (30).

### 2.10 Prediction of compound-protein binding affinity and molecular docking

To further explore the potential binding ability between the identified biomarkers and compounds, computational docking analysis was performed. Initially, the enrichR package (v 3.2) (31) was utilized to screen candidate compounds likely to interact with the biomarkers, based on the Drug Signatures Database (DSigDB) (https://dsigdb.tanlab.org/DSigDBv1.0/). Compounds with a P < 0.05 were selected as potential candidates. Following this, the interaction network linking compounds and biomarkers was depicted using Cytoscape software (v 3.10.1). Subsequently, compounds with high combined scores and non-hazardous properties were selected for molecular docking analysis. The AlphaFold database (https://alphafold.ebi.ac.uk/) was employed to predict the 3D structures of biomarker proteins, while the Protein Data Bank (PDB) database (https://www.rcsb.org/) was utilized to download their experimentally determined structures when available. The 3D structures of small-molecule compounds were retrieved from the public chemistry (PubChem) database (https://pubchem.ncbi.nlm.nih.gov/). Subsequently, the binding free energy between proteins and these compounds was assessed through molecular docking simulations on the Tamarind platform, with all input structures first prepared in PDBQT format.

### 2.11 Molecular dynamics simulations

Molecular dynamics simulations in GROMACS (v 2024.4) (32) with the AMBER99SB-ILDN force field and TIP3P water model were employed to evaluate the stability of compound-biomarker complexes following docking. The system, configured in a cubic box (1 nm protein-edge clearance) and neutralized with ions, underwent energy minimization via steepest descent, followed by NVT and NPT equilibration (300 K, V-rescale thermostat, 2 fs time step) for 100 ps. Finally, a 100 ns MD simulation was executed to calculate the root mean square deviation (RMSD), protein root mean square fluctuation (RMSF), total energy, and hydrogen bond count for the protein-compound complex. Finally, to quantitatively evaluate the binding affinity and thermodynamic stability of the protein-ligand complex, this study calculated the binding free energy using the Molecular Mechanics/Poisson-Boltzmann Surface Area (MM/PBSA) method based on stable conformational frames from the equilibrium phase of molecular dynamics simulations. The calculation formula is: $\Delta G_{bind}=G_{complex}-G_{receptor}-G_{ligand}$. All calculations were performed using the gmx_MMPBSA program, and the trend of binding free energy over simulation time was plotted using ggplot2.

### 2.12 Processing of scRNA-seq data and identification of crucial cell types

The Seurat package (v 5.1.0) (33) PercentageFeatureSet function was applied for QC on GSE289019. Filtering removed cells with <200 detected genes, genes present in <3 cells, mitochondrial content >15%, nFeature_RNA outside the 200–5,000 range, or nCount_RNA ≥ 20,000. To correct for batch effects, CCA was applied, and data were subsequently normalized using ScaleData. The contribution of principal components (PCs) was visualized with an ElbowPlot, and PCs at the inflection point were retained. Following the Seurat workflow, Uniform Manifold Approximation and Projection (UMAP)-based clustering at a resolution of 0.1 was conducted to identify distinct cell populations. Thereafter, annotation of cell clusters into specific cell types was performed using classical marker genes and prior literature (34, 35). A stacked bar chart visualizing the proportional distribution of cell types between RA and control groups was created with the ggplot2 package (v 3.5.2). Furthermore, the Wilcoxon test (P < 0.05) was applied to identify cell types in GSE289019 showing differential biomarker expression between RA and control samples. UMAP dimensionality reduction illustrated biomarker expression patterns across annotated cells. Cell populations demonstrating significant differential expression (P < 0.05) of several biomarkers were designated as crucial cell types. To further validate these findings, we examined the expression of these biomarkers in these cell types using the independent peripheral blood mononuclear cell data available in the CZ CELLxGENE Discover database (https://cellxgene.cziscience.com/).

### 2.13 Cellular communication and metabolism analyses

Using the CellChat package (v 1.6.1) (36) on GSE289019, we quantified both the count and magnitude of intercellular interactions among all annotated cell types in control versus RA samples. This analysis specifically sought to elucidate how the identified crucial cell types communicate with other cell populations in each group. Subsequently, the package was also employed to depict ligand-receptor interactions between annotated cell types and to quantify their communication likelihood. Additionally, variations in cellular metabolism between the RA and control groups were examined. By utilizing the scMetabolism package (v 0.2.1) (37), metabolic pathways in annotated cell types were scored in combination with VISION, AUCell, ssGSEA, and GSVA algorithms. Ultimately, activity scores for cells within each metabolic pathway were obtained. The VISION algorithm was applied to evaluate differences in metabolic pathway activity among cell types, scoring the enrichment of KEGG metabolic pathways in crucial cell populations.

### 2.14 Pseudo-temporal trajectory analysis and evaluating TF activities

The investigation of heterogeneity and differentiation trajectories in crucial cell types commenced with dimensionality reduction clustering analysis. The Seurat package (v 5.0.1) clustering function was employed, with a resolution parameter of 0.1, which resulted in the re-clustering of these crucial cell types into distinct subclusters. Subsequently, UMAP was employed for cell clustering. Following this, marker gene information from the references (34, 38) was utilized to annotate each cell subcluster into distinct cell subpopulations. Thereafter, the monocle package (v 2.30.1) (39) was utilized to perform pseudo-temporal trajectory analysis on crucial cell types, aiming to simulate cellular differentiation dynamics and developmental processes. Additionally, dynamic expression patterns of biomarkers across the differentiation course of key cell types were delineated using the monocle package (v 2.30.1).Further analysis was conducted to identify key regulators in these crucial cell subpopulations. Specifically, the pySCENIC tool (https://github.com/aertslab/pySCENIC) was employed to predict TFs and their downstream target genes within these subpopulations. Subsequently, the SCENIC package (v 1.3.1) (40) was utilized to construct gene regulatory networks (GRNs). Following this, the AUCell method was applied to calculate specificity scores of TFs across different cell subpopulations, and the top 5 key regulators were selected based on their rankings to determine the core regulatory factors in each subpopulation.

### 2.15 Cell cycle analysis and gene set variation analysis

To investigate cell cycle dynamics during differentiation, the CellCycleScoring function from the Seurat package (v 5.0.1) was employed. By evaluating S, G2, and M phase scores in crucial cell populations based on marker gene expression patterns, the cell cycle stages of various cell types were inferred. Moreover, the GSVA package (v 1.50.0) (29) was employed to compute GSVA scores for all GSE289019 samples, enabling a comprehensive characterization of pathway activity differences between the RA and control groups. Reference gene sets (c2.cp.kegg_medicus.v2025.1.Hs.symbols.gmt) were obtained from MSigDB. Differential pathway activity between RA and control groups was then assessed using the limma package (v 3.58.1), applying thresholds of |t| > 2 and P < 0.05.

### 2.16 Ethics Statement

This study was conducted in accordance with the Declaration of Helsinki and approved by the Biomedical Ethics Committee of the 900th Hospital of Joint Logistic Support Forces (Approval No. 2025-169). All participants provided written informed consent prior to enrollment. In this study, synovial tissue was obtained from 3 patients with RA who underwent total knee arthroplasty and 3 patients who underwent meniscal repair as controls; peripheral blood samples were collected from 8 patients with RA and 8 age- and sex-matched healthy volunteers.

### 2.17 Reverse transcription-quantitative PCR

Peripheral blood was collected from 5 patients with RA and 5 healthy controls, and PBMCs were separated from venous blood by Ficoll-Paque density gradient centrifugation. Total RNA was isolated with the Steady Pure Rapid RNA Extraction Kit, followed by genomic DNA removal and cDNA synthesis using a commercial kit. Real-time quantitative PCR employed SYBR Green Master Mix (Servicebio, G3328-05) on a PCR detection system. Primers for CD74, PGLYRP1, TXN, and β-actin (designed via Primer-BLAST and synthesized by General Biotech Co., Ltd.) were used. Relative mRNA levels were determined by the 2^-ΔΔCt^ method normalized to β-actin, with all reactions run in triplicate. GraphPad Prism software was employed for statistical evaluation. The primer sequences used are listed in **Table 1**.

**Table 1:** The primer sequences.

Gene	Forward primer (5′→3′)	Reverse primer (5′→3′)
CD74	GGCAACATGACAGAGGACCA	GCTCTCACATGGGGACTGG
PGLYRP1	TGGGCAACTACATGGATCGG	CACATCCCGGTGTCCTTTGA
TXN	AGCAGATCGAGAGCAAGACTG	AGCAACATCATGAAAGAAAGGCT
β-actin	CATGTACGTTGCTATCCAGGC	CTCCTTAATGTCACGCACGAT

### 2.18 Western blotting

After PBMC cell collection (3 from the RA group and 3 from the control group), the cells were lysed on ice using RIPA strong lysis buffer (Servicebio, G2002) in combination with protease inhibitors (Proteintech, PR20032). Subsequently, After centrifugation (4 °C, 16,000 g, 15 min), the supernatant was isolated and protein content quantified using a BCA kit (Beyotime, P0009). Equal amounts of protein were mixed with 5× loading buffer (Servicebio, G2013) and denatured by boiling at 100 °C for 10 minutes. Subsequently, SDS-PAGE electrophoresis was performed (Three biological replicates per group; no technical replicates were performed). A 10%-12% resolving gel was prepared according to the molecular weight of the target protein. After electrophoresis, the proteins were electroblotted onto a PVDF membrane (Millipore, 0.45 μm). After TBST washes, non-specific binding was blocked with 5% BSA or skim milk. The membrane was incubated overnight at 4 °C with primary antibodies specific for CD74, PGLYRP1, TXN, and β-actin (obtained from Servicebio, Huamei Biotechnology, and Proteintech). Following TBST washes, an HRP-conjugated secondary antibody (Servicebio) was applied. Detection was performed with ECL chemiluminescence substrate, and images were captured.

### 2.19 Tissue chip collection and immunohistochemical staining

Synovial tissue sections embedded in paraffin (3 cases in the RA group and 3 cases in the control group) were selected, with one section taken from each case, and baked at 64 °C for 1 hour. Subsequently, tissue sections were deparaffinized with xylene (Xilong Scientific) and rehydrated through descending ethanol concentrations, then washed with PBS (Biosharp). High-pressure antigen retrieval was performed using citrate buffer (Servicebio, G1210). Following cooling, endogenous peroxidase was inactivated with 3% H_2_O_2_ (20 min, room temperature), and sections were subsequently blocked with 5% bovine serum albumin (Servicebio) at 37 °C for 30 min. Subsequently, primary antibodies against PGLYRP1 (CUSABIO, CSB-PA017862, 1:200), TXN (Servicebio, GB11993-50, 1:200), and CD74 (Servicebio, GB115427-50, 1:200) were added respectively. The sections were incubated overnight at 4 °C. After warming up, secondary antibody incubation was carried out using a Universal Two-Step Detection Kit (Zhongshan Golden Bridge, PV-9000). The sections were then visualized with a DAB Chromogenic Kit (Zhongshan Golden Bridge, ZLI-9019), and the nuclei were counterstained with hematoxylin (Servicebio). Following dehydration through a gradient of alcohol solutions and clearing in xylene, the sections were mounted with neutral balsam. Finally, the entire slide was scanned, and three fields of view were randomly selected from each section for semi-quantitative analysis.

### 2.20 Statistical analysis

R software (v 4.3.1) was used for statistical analyses, applying the Wilcoxon test for group comparisons. In the experimental section, data are presented as mean ± standard deviation (Mean ± SD). Normality was assessed using the Shapiro-Wilk test, with p > 0.05 indicating a normal distribution. Homogeneity of variances was assessed using the F-test, with p > 0.05 indicating homogeneity of variances. When comparing two groups, a t-test was used if the data were both normally distributed and had homogeneous variances; otherwise, the Wilcoxon test was used.

## 3 Results

### 3.1 Identification and functional analysis of candidate genes associated with RA

**Figure 1:**
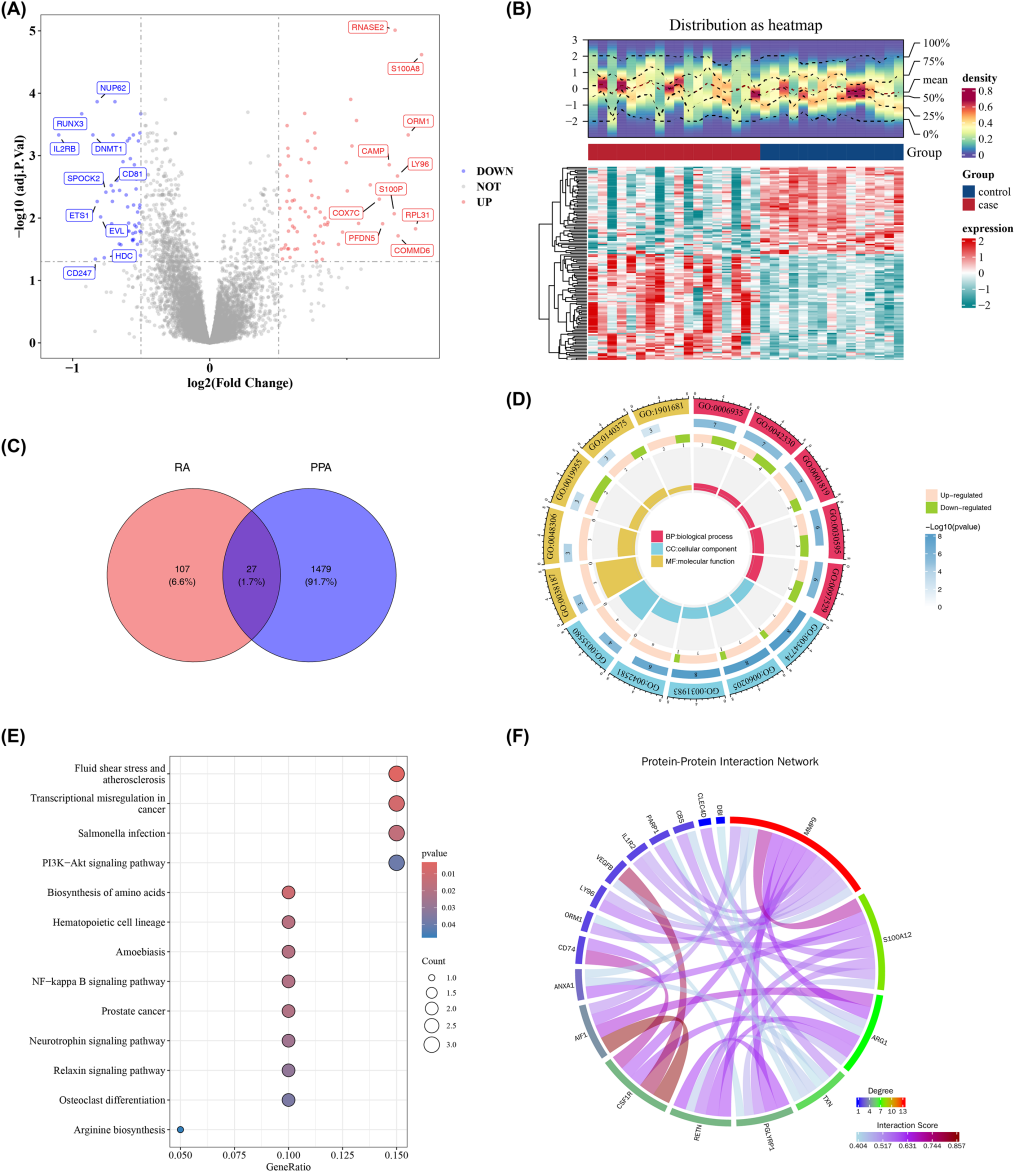
Identification and functional analysis of candidate genes. **(A)** Volcano plot illustrating the differential expression of genes between the RA and control groups. Downregulated genes are shown in blue, while upregulated genes are in red. **(B)** Heatmap displaying the expression profiles of DEGs between RA and control groups. Gene expression is represented with a color gradient from red (high) to green (low). **(C)** Venn diagram showing the overlap between the DEGs and the 1,506 RA-associated protein-coding genes, leading to the identification of 27 candidate genes. **(D)** Circular plot representing the enrichment of GO terms for the 27 candidate genes, categorized into BP, CC, and MF. **(E)** KEGG pathway analysis of the 27 candidate genes. **(F)** PPI network for the candidate genes.

A total of 134 DEGs were identified in RA versus control comparisons, including 74 downregulated and 60 upregulated genes (**Figures 1A, B**). By conducting a screening process to identify the intersection between the DEGs and a set of 1,506 protein-coding genes associated with RA, 27 candidate genes were ultimately identified (**Figure 1C**). A total of 631 GO terms showed significant enrichment for the 27 candidate genes (P < 0.05), including 556 BPs, 33 CCs, and 42 MFs (**Supplementary Table 2**). Specifically, the candidate genes were primarily associated with BPs such as “chemotaxis,” “taxis,” and “positive regulation of cytokine production” (**Figure 1D**). In terms of CCs, they were closely linked to structures like the “secretory granule lumen” and “cytoplasmic vesicle lumen”. Regarding MFs, they were involved in activities including “pattern recognition receptor activity,” “calcium-dependent protein binding,” and “cytokine binding”. Furthermore, The candidate genes showed significant involvement in 13 KEGG pathways (P < 0.05), notably including “biosynthesis of amino acids” and the “NF-kappa B signaling pathway” (**Figure 1E**; **Supplementary Table 3**). The 27 candidate genes were found to play key roles in RA immunity, mainly by modulating immune cell trafficking, cytokine production, and the activation of inflammatory signaling cascades. Furthermore, during the protein-level interaction analysis of the 27 candidate genes, nine proteins were isolated with no interactions, while the other 18 exhibited mutual interactions (**Figure 1F**). These 18 genes were identified as hub genes. Notably, the MMP9 protein demonstrated the highest level of connectivity with other proteins.

### 3.2 CD74, PGLYRP1, and TXN were identified as biomarkers

**Figure 2:**
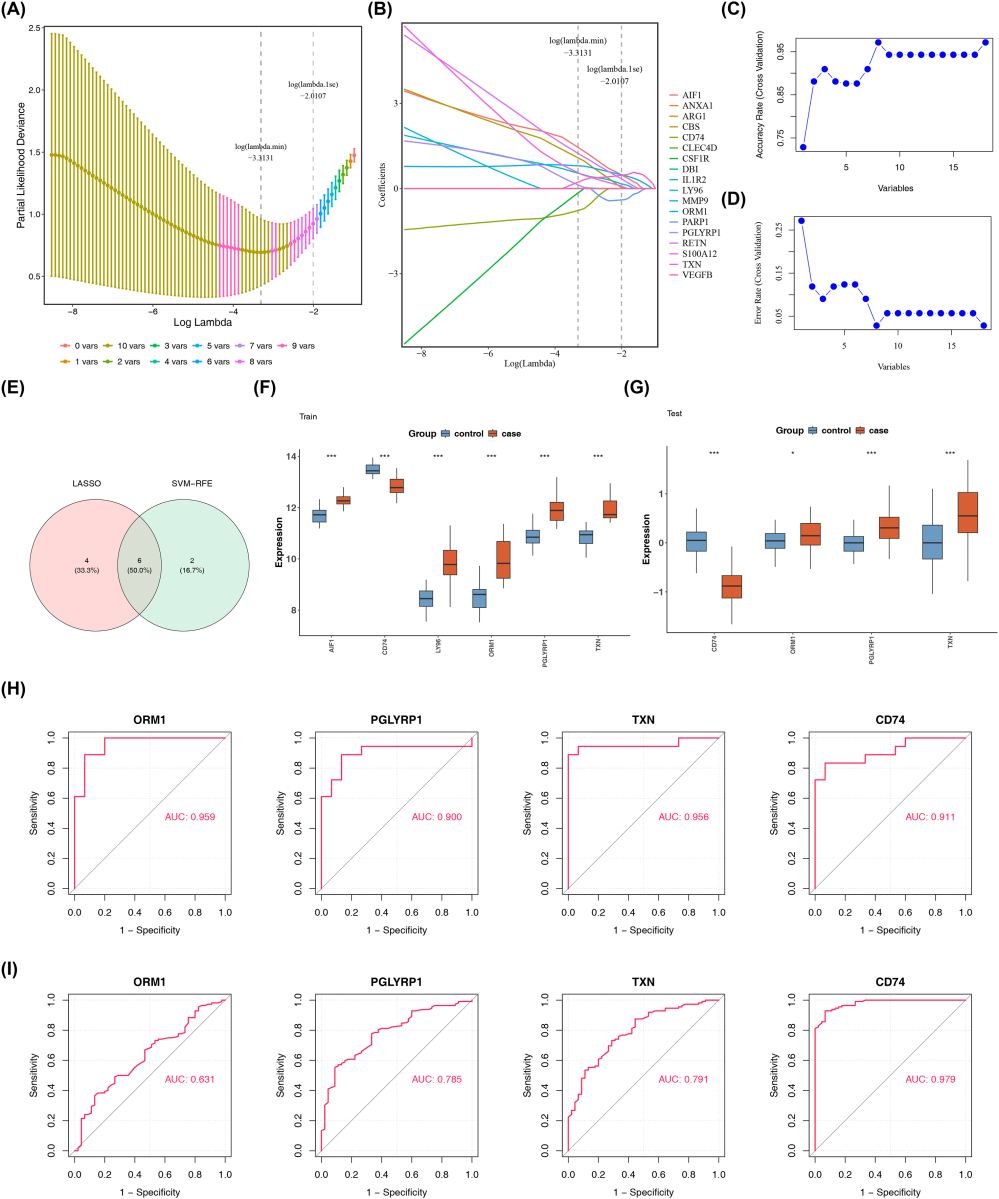
Identification of biomarkers for RA. **(A)** LASSO regression analysis for gene selection. The plot shows partial likelihood deviance as a function of log(lambda), with the optimal lambda (log(lambda.min) = -3.3131) chosen based on the lowest error rate. **(B)**Coefficients of the genes selected by LASSO regression analysis. **(C)** SVM-RFE algorithm showing the accuracy rate of model performance across different numbers of variables. **(D)** SVM-RFE error rate plot indicating the minimum error at a specific number of variables. **(E)** Venn diagram illustrating the intersection of genes identified by LASSO regression and SVM-RFE, resulting in six common characteristic genes (PGLYRP1, CD74, ORM1, LY96, TXN, and AIF1). **(F)** Boxplot showing the expression profiles of common characteristic genes in GSE15573. ***P < 0.001. **(G)** Boxplot showing the expression profiles of common characteristic genes in GSE17755. *P < 0.05; ***P < 0.001. **(H)** ROC curve analysis for ORM1, CD74, PGLYRP1, and TXN in GSE15573. **(I)** ROC curve analysis for ORM1, CD74, PGLYRP1, and TXN in GSE17755.

LASSO regression identified 10 characteristic genes (CD74, AIF1, CSF1R, PGLYRP1, TXN, ORM1, LY96, RETN, CBS, and S100A12) at the optimal lambda value (log(lambda.min) = -3.3131) corresponding to the lowest model error rate (**Figures 2A, B**). Additionally, by applying the SVM-RFE algorithm, the number of variables corresponding to the point where the minimum error was first achieved was selected, which resulted in the identification of eight genes (**Figures 2C, D**). These genes were determined as SVM-RFE characteristic genes, namely ORM1, TXN, PARP1, ARG1, CD74, AIF1, LY96, and PGLYRP1. The intersection of genes identified by LASSO regression and SVM-RFE methods yielded six common characteristic genes (PGLYRP1, CD74, ORM1, LY96, TXN, and AIF1) (**Figure 2E**). Expression validation in GSE15573 and GSE17755 showed that ORM1, PGLYRP1, and TXN were significantly upregulated (P < 0.05) in both datasets, whereas CD74 was significantly downregulated (P < 0.001) (**Figures 2F, G**). AIF1 and LY96 were not detected in GSE17755. CD74, PGLYRP1, and TXN each yielded AUC values > 0.7 in GSE15573 and GSE17755 (**Figures 2H, I**), indicating robust diagnostic performance with good sensitivity and specificity for distinguishing RA from control samples. Therefore, based on the combined assessment of expression differences and diagnostic value, CD74, PGLYRP1, and TXN were ultimately identified as biomarkers for RA.

### 3.3 Nomogram construction, subcellular localization, and tissue/organ expression of CD74, PGLYRP1, and TXN

**Figure 3:**
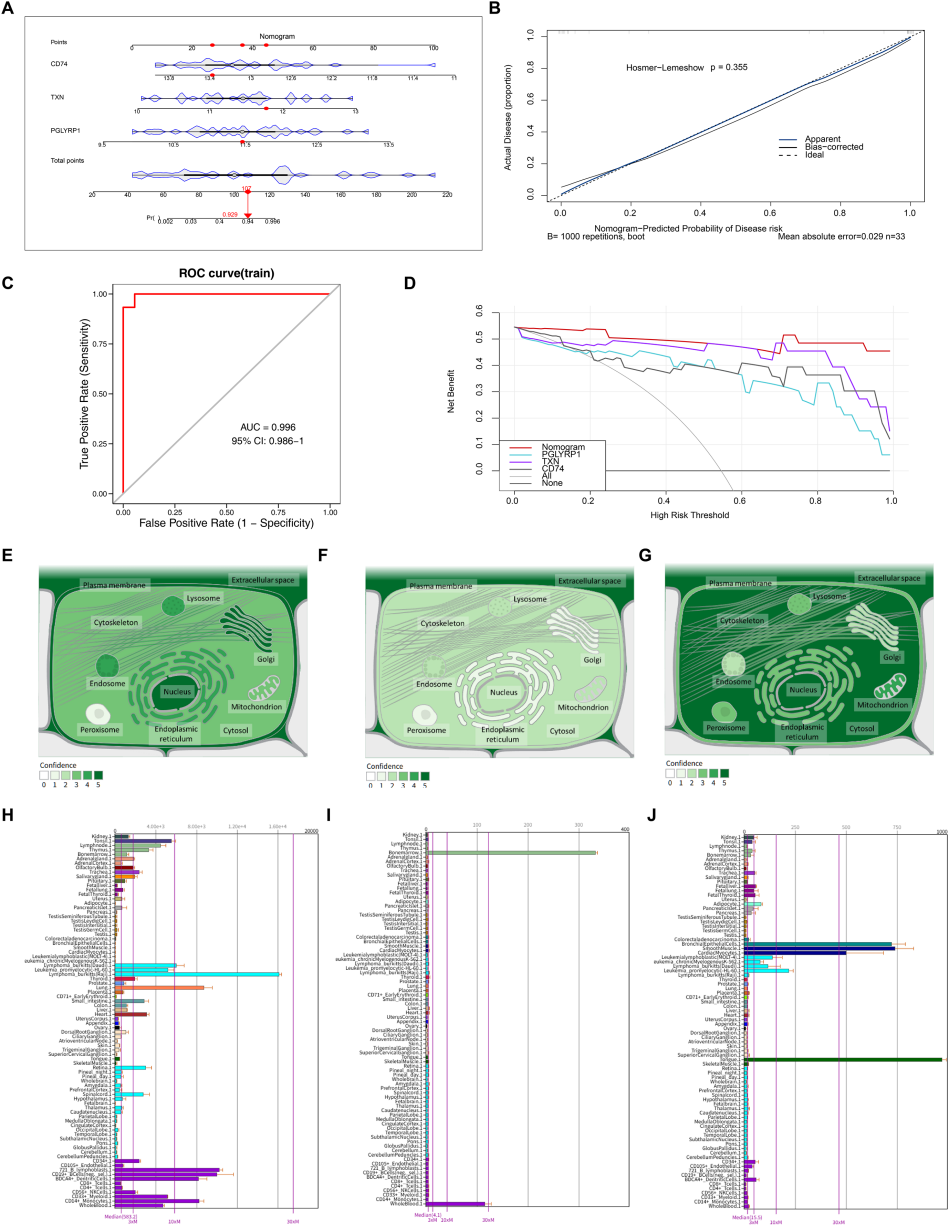
Nomogram model and functional characterization of RA biomarkers. **(A)** Nomogram model based on the expression of CD74, PGLYRP1, and TXN in GSE15573 to predict the diagnostic probability of RA. The line segments correspond to the contribution of each biomarker to the final score. **(B)** Calibration curve analysis of the nomogram model, showing the predicted probability versus actual disease status. **(C)** ROC curve analysis of the nomogram model, with an AUC value of 0.996. **(D)** DCA comparing the nomogram model with standalone biomarkers (PGLYRP1, TXN, CD74). **(E)** Subcellular localization of CD74 in the Golgi apparatus, nucleus, and extracellular space, as visualized through cellular imaging. **(F)** Subcellular localization of PGLYRP1 primarily in the extracellular space, based on imaging data. **(G)** Subcellular localization of TXN in the cytosol, nucleus, and extracellular space, as shown through cellular imaging. **(H)** Tissue-specific expression of CD74 based on the BioGPS database. **(I)** Tissue-specific expression of PGLYRP1. **(J)** Tissue-specific expression of TXN.

Based on CD74, PGLYRP1, and TXN expression in GSE15573, a nomogram was developed to estimate RA diagnostic probability. The contribution of each biomarker to the outcome was reflected by its corresponding line segment length, and the scoring system allowed quantification of individual variable contributions and overall risk prediction. For instance, when the red dots corresponding to the expressions of CD74, PGLYRP1, and TXN were selected, the total points amounted to 107, and the predicted probability of disease was 92.9% (**Figure 3A**). Good model fit was confirmed by calibration curve evaluation (**Figure 3B**) and the HL test (P = 0.355). Additionally, ROC analysis revealed an AUC of 0.996, reflecting good predictive accuracy (**Figure 3C**). In addition, when contrasted with the utilization of PGLYRP1, TXN, or CD74 as standalone agents, the nomogram model demonstrated a markedly superior predictive capacity (**Figure 3D**). It was evident that, particularly at higher risk thresholds, the net benefit of the nomogram remained consistently high. Consequently, the DCA further substantiated the favorable clinical utility of the nomogram model in disease prediction. It is worth noting that in the validation set GSE17755, this nomogram also demonstrated good predictive performance, with an AUC of 0.972 and a non-significant Hosmer-Lemeshow test result (P = 0.989) (**Supplementary Figures 1A, B**).

Further analysis of the subcellular localization of CD74, PGLYRP1, and TXN revealed that CD74 was primarily localized in the Golgi apparatus, nucleus, and extracellular space (**Figure 3E**); PGLYRP1 was mainly distributed extracellularly (**Figure 3F**); and TXN was predominantly found in the cytosol, nucleus, and extracellular space (**Figure 3G**). This localization information provided crucial clues for a deeper understanding of the functions of these biomarkers within cells. Moreover, analysis of the BioGPS database revealed marked variations in the mRNA expression levels of CD74, PGLYRP1, and TXN across diverse tissues. **Table 2** presents the GO functional annotations of the CD74, PGLYRP1, and TXN genes, further elucidating their roles in BPs, CCs, and MFs. The CD74 exhibited a strong expression preference in tissues related to the human immune system, particularly in B lymphoblasts (**Figure 3H**). The PGLYRP1 showed high expression levels in bone marrow and whole blood (**Figure 3I**). The TXN demonstrated relatively high expression in smooth muscle and bronchial epithelial cells (**Figure 3J**). The tissue-specific expression patterns of these biomarkers offered clues regarding their potential roles in RA.

**Table 2:** GO functional annotation results of biomarkers from the BioGPS database.

Gene name	Symbol	Function
thioredoxin	TXN	Molecular Function RNA binding (GO:0003723) thioredoxin-disulfide reductase (NADPH) activity (GO:0004791) protein binding (GO:0005515) protein-disulfide reductase activity (GO:0015035) protein homodimerization activity (GO:0042803) protein-disulfide reductase [NAD(P)H] activity (GO:0047134) Biological Process negative regulation of transcription by RNA polymerase II (GO:0000122) response to radiation (GO:0009314) cellular homeostasis (GO:0019725) positive regulation of DNA binding (GO:0043388) cell redox homeostasis (GO:0045454) negative regulation of protein export from nucleus (GO:0046826) positive regulation of phosphatidylinositol 3-kinase/protein kinase B signal transduction (GO:0051897) cellular detoxification of hydrogen peroxide (GO:0061692) response to nitric oxide (GO:0071731) Cellular Component extracellular region (GO:0005576) nucleus (GO:0005634) nucleoplasm (GO:0005654) cytoplasm (GO:0005737) cytosol (GO:0005829) extracellular exosome (GO:0070062)
CD74 molecule	CD74	Molecular Function amyloid-beta binding (GO:0001540) cytokine receptor activity (GO:0004896) protein binding (GO:0005515) cytokine binding (GO:0019955) MHC class II protein complex binding (GO:0023026) macrophage migration inhibitory factor binding (GO:0035718) MHC class II protein binding (GO:0042289) CD4 receptor binding (GO:0042609) MHC class II protein binding, via antigen binding groove (GO:0042658) identical protein binding (GO:0042802) protein folding chaperone (GO:0044183) nitric-oxide synthase binding (GO:0050998) Biological Process prostaglandin biosynthetic process (GO:0001516) positive regulation of protein phosphorylation (GO:0001934) positive regulation of cytokine-mediated signaling pathway (GO:0001961) adaptive immune response (GO:0002250) T cell activation involved in immune response (GO:0002286) immune system process (GO:0002376) positive regulation of dendritic cell antigen processing and presentation (GO:0002606) negative regulation of peptide secretion (GO:0002792) positive regulation of type 2 immune response (GO:0002830) negative regulation of mature B cell apoptotic process (GO:0002906) protein folding (GO:0006457) intracellular protein transport (GO:0006886) defense response (GO:0006952) immune response (GO:0006955) positive regulation of gene expression (GO:0010628) immunoglobulin mediated immune response (GO:0016064) antigen processing and presentation (GO:0019882) antigen processing and presentation of endogenous antigen (GO:0019883) antigen processing and presentation of exogenous peptide antigen via MHC class II (GO:0019886) negative regulation of cell migration (GO:0030336) positive regulation of B cell proliferation (GO:0030890) positive regulation of prostaglandin biosynthetic process (GO:0031394) positive regulation of chemokine production (GO:0032722) positive regulation of interleukin-6 production (GO:0032755) positive regulation of interleukin-8 production (GO:0032757) positive regulation of kinase activity (GO:0033674) response to type II interferon (GO:0034341) macrophage migration inhibitory factor signaling pathway (GO:0035691) regulation of macrophage activation (GO:0043030) negative regulation of apoptotic process (GO:0043066) positive regulation of canonical NF-kappaB signal transduction (GO:0043123) positive regulation of MAPK cascade (GO:0043410) negative regulation of DNA damage response, signal transduction by p53 class mediator (GO:0043518) T cell selection (GO:0045058) positive thymic T cell selection (GO:0045059) negative thymic T cell selection (GO:0045060) negative regulation of T cell differentiation (GO:0045581) positive regulation of T cell differentiation (GO:0045582) positive regulation of monocyte differentiation (GO:0045657) positive regulation of DNA-templated transcription (GO:0045893) host-mediated suppression of symbiont invasion (GO:0046597) positive regulation of viral entry into host cell (GO:0046598) positive regulation of fibroblast proliferation (GO:0048146) protein stabilization (GO:0050821) positive regulation of macrophage cytokine production (GO:0060907) protein-containing complex assembly (GO:0065003) protein trimerization (GO:0070206) positive regulation of ERK1 and ERK2 cascade (GO:0070374) positive regulation of neutrophil chemotaxis (GO:0090023) negative regulation of intrinsic apoptotic signaling pathway in response to DNA damage by p53 class mediator (GO:1902166) positive regulation of chemokine (C-X-C motif) ligand 2 production (GO:2000343) positive regulation of macrophage migration inhibitory factor signaling pathway (GO:2000448) Cellular Component Golgi membrane (GO:0000139) extracellular region (GO:0005576) nucleus (GO:0005634) cytoplasm (GO:0005737) lysosome (GO:0005764) lysosomal membrane (GO:0005765) endosome (GO:0005768) late endosome (GO:0005770) multivesicular body (GO:0005771) vacuole (GO:0005773) endoplasmic reticulum (GO:0005783) endoplasmic reticulum membrane (GO:0005789) Golgi apparatus (GO:0005794) plasma membrane (GO:0005886) external side of plasma membrane (GO:0009897) cell surface (GO:0009986) ER to Golgi transport vesicle membrane (GO:0012507) membrane (GO:0016020) transport vesicle membrane (GO:0030658) endocytic vesicle membrane (GO:0030666) clathrin-coated endocytic vesicle membrane (GO:0030669) trans-Golgi network membrane (GO:0032588) protein-containing complex (GO:0032991) macrophage migration inhibitory factor receptor complex (GO:0035692) NOS2-CD74 complex (GO:0035693) MHC class II protein complex (GO:0042613) lysosomal lumen (GO:0043202) extracellular exosome (GO:0070062) lumenal side of endoplasmic reticulum membrane (GO:0098553)
peptidoglycan recognition protein 1	PGLYRP1	Molecular Function zinc ion binding (GO:0008270) N-acetylmuramoyl-L-alanine amidase activity (GO:0008745) peptidoglycan immune receptor activity (GO:0016019) Hsp70 protein binding (GO:0030544) receptor ligand activity (GO:0048018) molecular adaptor activity (GO:0060090) Biological Process immune system process (GO:0002376) immune response (GO:0006955) signal transduction (GO:0007165) peptidoglycan catabolic process (GO:0009253) response to bacterium (GO:0009617) detection of bacterium (GO:0016045) killing of cells of another organism (GO:0031640) negative regulation of type II interferon production (GO:0032689) negative regulation of natural killer cell differentiation involved in immune response (GO:0032827) defense response to bacterium (GO:0042742) innate immune response (GO:0045087) negative regulation of inflammatory response (GO:0050728) defense response to Gram-positive bacterium (GO:0050830) biological process involved in interaction with host (GO:0051701) antimicrobial humoral immune response mediated by antimicrobial peptide (GO:0061844) Cellular Component extracellular region (GO:0005576) extracellular space (GO:0005615) specific granule lumen (GO:0035580) extracellular exosome (GO:0070062) phagocytic vesicle lumen (GO:0097013) tertiary granule lumen (GO:1904724)

### 3.4 Functional analysis for CD74, PGLYRP1, and TXN

**Figure 4:**
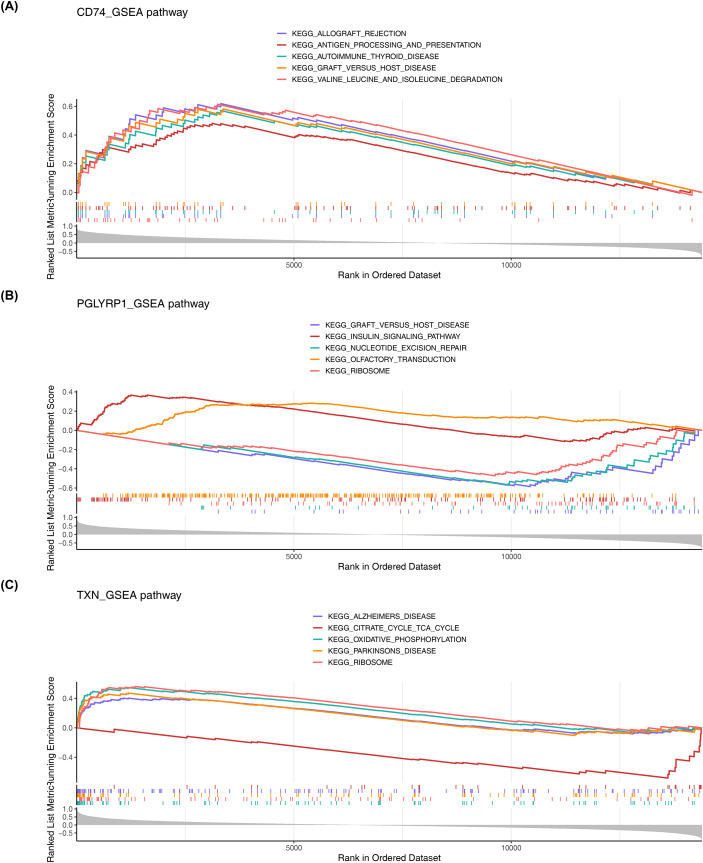
GSEA pathway analysis of RA biomarkers. **(A)** GSEA pathway analysis for CD74. **(B)** GSEA pathway analysis for PGLYRP1. **(C)** GSEA pathway analysis for TXN.

Subsequent exploration was conducted via GSEA to examine the enrichment patterns of signaling pathways associated with CD74, PGLYRP1, and TXN. The pathways enriched for CD74 included “allograft rejection”, which was closely linked to immune system dysregulation (**Figure 4A**). Additionally, CD74 was associated with the “antigen processing and presentation pathway”, suggesting its possible involvement in immune tolerance and activation. The analysis revealed that PGLYRP1 was enriched in pathways such as “olfactory transduction” (**Figure 4B**). This pathway was closely associated with immune responses, suggesting that PGLYRP1 might have played a role in the immune-mediated processes of RA. Enrichment pathway analysis for TXN demonstrated its association with functions related to “oxidative phosphorylation” and “ribosome” (**Figure 4C**). These pathways were closely linked to cellular energy metabolism and protein synthesis. In RA studies, TXN might have influenced the onset and progression of arthritis through redox regulation. Collectively, the enrichment of CD74, PGLYRP1, and TXN in key pathways related to immune responses, inflammatory processes, and redox reactions unveiled their potential roles in RA.

### 3.5 Immune cells distribution and correlation analysis of CD74, PGLYRP1, and TXN

**Figure 5:**
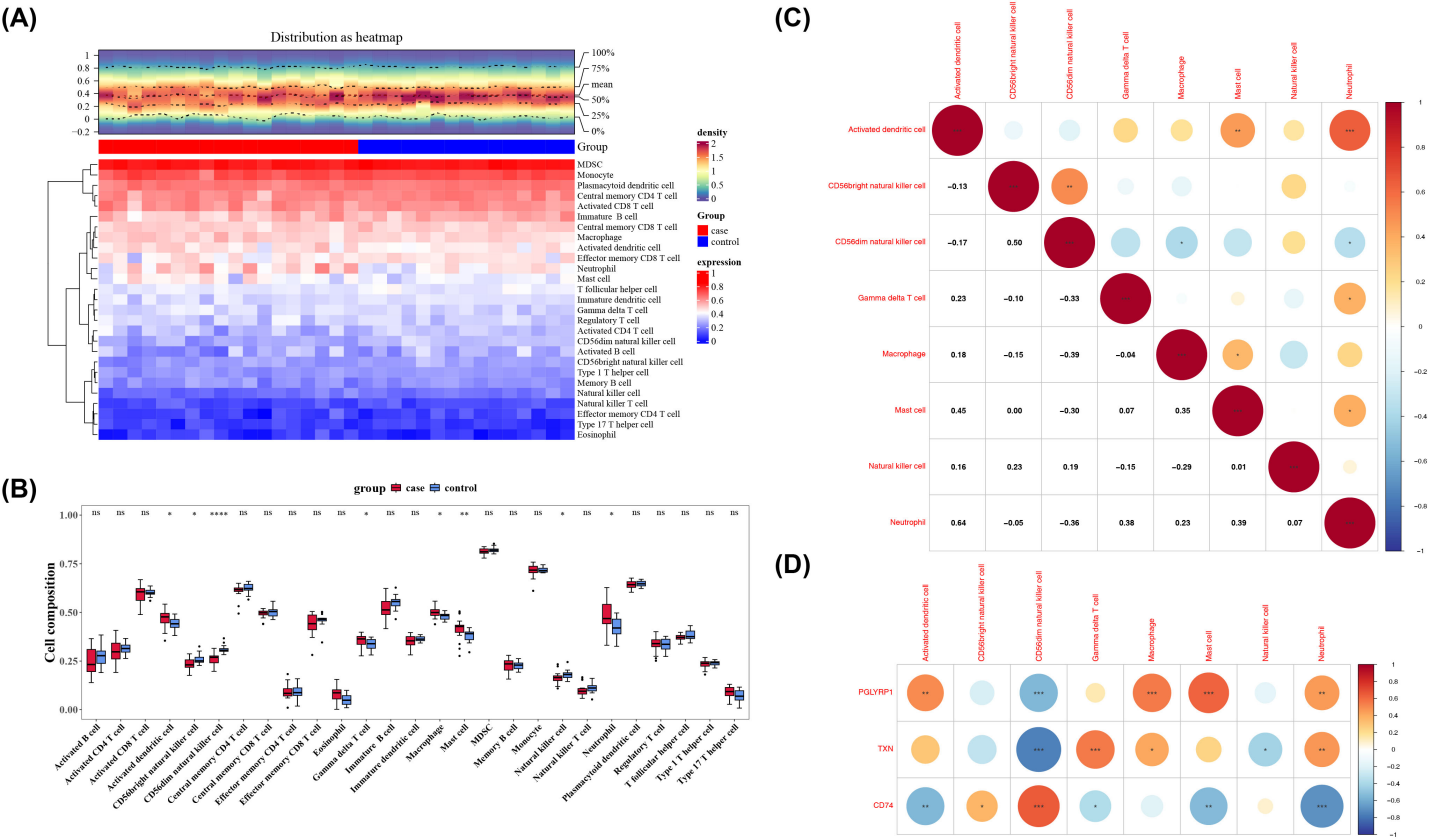
Immune cell distribution and correlation analysis of RA biomarkers. **(A)** Heatmap depicting the relative abundance of 28 immune cell types in the RA and control groups from GSE15573, analyzed using the ssGSEA method. **(B)** Boxplot comparing the immune cell infiltration levels between the RA and control groups for eight significantly different immune cell types. *P < 0.05; **P < 0.01; ****P < 0.0001; ns, not significant. **(C)** Correlation matrix showing the relationships among differentially infiltrated immune cell types. *P < 0.05; **P < 0.01; ***P < 0.001; ****P < 0.0001. **(D)** Correlation analysis between differentially infiltrated immune cell types and the RA biomarkers CD74, PGLYRP1, and TXN. *P < 0.05; **P < 0.01; ***P < 0.001; ****P < 0.0001.

Relative abundances of 28 immune cell types in GSE15573 were estimated via ssGSEA, revealing MDSCs as the most abundant population (**Figure 5A**). Eight cell types showed significantly different infiltration between RA and controls (**Figure 5B**). Key correlations included a positive association between activated dendritic cells and neutrophils (cor = 0.64, P < 0.001) and a negative association between macrophages and CD56dim NK cells (cor = -0.39, P < 0.05) (**Figure 5C**). Notably, CD56dim NK cells were strongly positively correlated with CD74 (cor = 0.66, P < 0.001) and strongly negatively correlated with TXN (cor = -0.75, P < 0.001) (**Figure 5D**; **Supplementary Table 4**), suggesting a potential association between CD74/TXN and CD56dim NK cells that may warrant further investigation into their possible roles in RA immune regulation.

### 3.6 Molecular regulatory network and drug interactions involving CD74, PGLYRP1, and TXN

**Figure 6:**
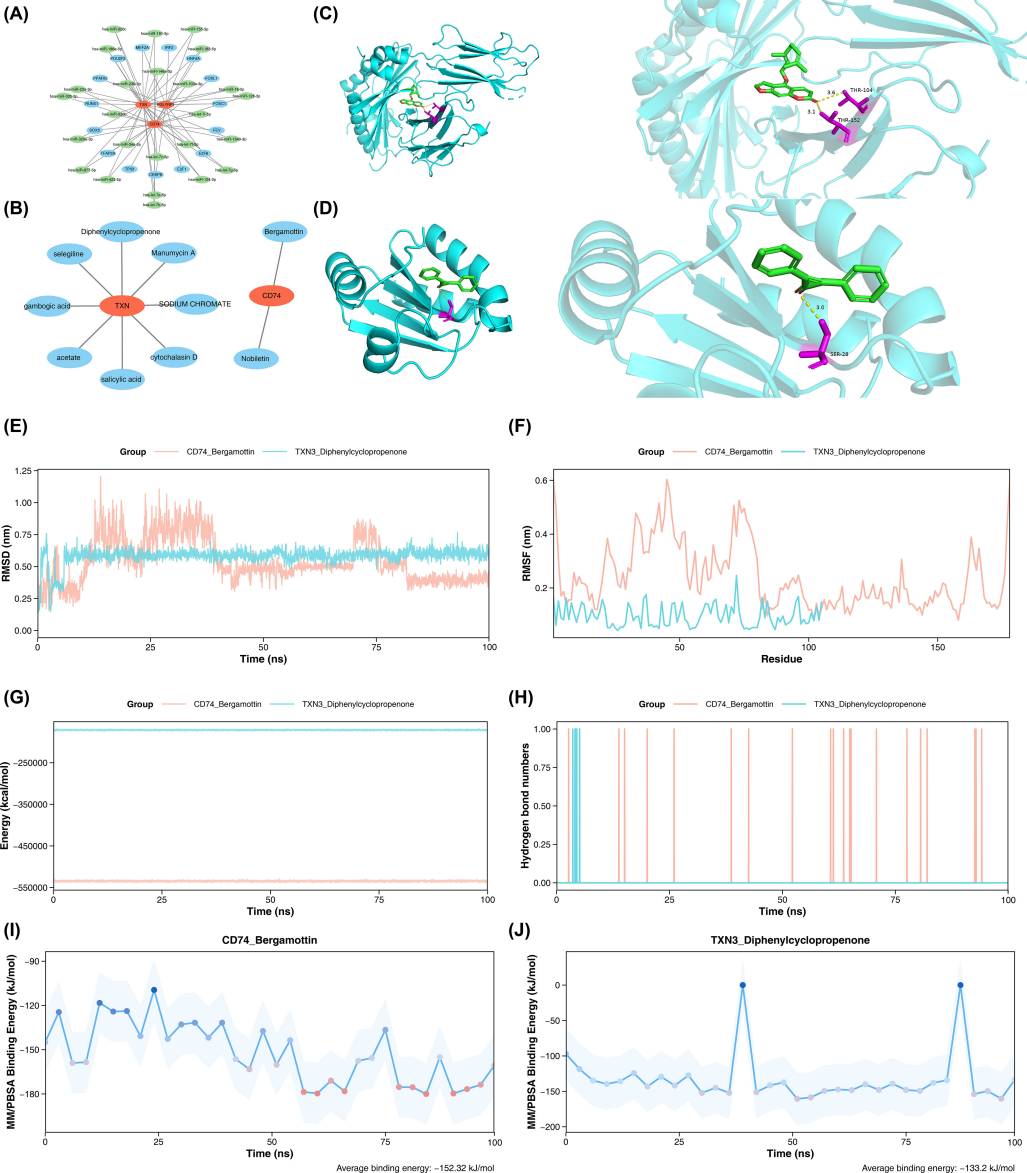
miRNA, TFs, drug targeting, and molecular docking of RA biomarkers. **(A)** Network of miRNAs and TFs predicted to target CD74, PGLYRP1, and TXN. **(B)** Top 10 potential drugs identified for CD74, PGLYRP1, and TXN based on their combined score. **(C)** Molecular docking of CD74 with bergamottin. **(D)** Molecular docking of TXN with diphenylcyclopropenone. **(E)** RMSD plot for the stability of CD74-bergamottin and TXN-diphenylcyclopropenone complexes over time. **(F)** RMSF plot showing the flexibility of the residues in the CD74-bergamottin and TXN-diphenylcyclopropenone complexes. **(G)** Energy plot illustrating the energy variations over time for the CD74-bergamottin and TXN-diphenylcyclopropenone complexes. **(H)** Hydrogen bond plot for CD74-bergamottin and TXN-diphenylcyclopropenone complexes. **(I–J)** MM/PBSA calculations revealed that the average binding free energies for CD74-Bergamottin **(I)** and TXN-Diphenylcyclopropenone **(J)** were -152.32 and -133.2 kJ/mol, respectively, indicating stable complex formation.

Further exploration was conducted into the roles of miRNAs and TFs in regulating CD74, PGLYRP1, and TXN. The results revealed that 61, three, and 94 miRNAs were predicted to target CD74, PGLYRP1, and TXN, respectively (**Supplementary Table 5**). Ultimately, 25 miRNAs that concurrently regulated two or more of these biomarkers were selected as key miRNAs. For instance, CD74 and TXN were jointly regulated by hsa-miR-146a-5p, while PGLYRP1 and TXN were co-regulated by hsa-miR-155-5p. Additionally, 15 TFs were identified. Among them, five TFs (e.g., FEV) were linked to CD74, eight TFs (e.g., PPARG) to PGLYRP1, and four TFs (e.g., POU2F2) to TXN (**Figure 6A**).

Further screening was conducted to identify candidate compounds potentially targeting CD74, PGLYRP1, and TXN. A total of 161 meaningful compounds were identified, with five compounds predicted for CD74, 157 for TXN, and none for PGLYRP1 (**Supplementary Table 6**). The top 10 compounds based on the combined score are shown (**Figure 6B**). Subsequently, molecular docking was performed between CD74 and bergamottin, as well as between TXN and diphenylcyclopropenone, with binding free energies of -6.240 and -5.736 kcal/mol (**Table 3**), respectively. The relevant docking images are shown in **Figures 6C, D**. Binding affinities were evaluated using a threshold of -5 kcal/mol, below which interactions were deemed strong. On this basis, both CD74-bergamottin and TXN-diphenylcyclopropenone exhibited favorable binding characteristics in silico. To provide additional computational validation for the credibility of molecular docking, MDs were conducted. The RMSD serves as a dependable metric for assessing the conformational stability of protein–ligand complexes by measuring the degree of atomic displacement from their original positions. In MDs, when a protein’s RMSD stabilizes, it indicates that the system is approaching an equilibrium conformation, thereby acting as a crucial criterion for evaluating the stability of the simulation. The RMSD fluctuations of the CD74-bergamottin and TXN-diphenylcyclopropenone complexes were relatively small (**Figure 6E**), indicating stable binding between the compound and protein, with no significant conformational changes in the complexes and robust interactions between the compound and protein. The RMSF measures the variability of each amino acid’s position throughout the simulation, with lower RMSF values denoting more rigid regions and higher values indicating greater flexibility. Bergamottin likely interacted with the flexible region of the CD74 protein (such as the 50–70 residue segment) to form a stable binding, while other regions exhibited structural rigidity, contributing to the overall stability of the complex (**Figure 6F**). The fluctuations of the TXN-Diphenylcyclopropenone complex were primarily concentrated in the residue segments of 50–90 and 150-170, indicating that these regions might be the compound-binding sites. Other regions displayed strong rigidity, contributing to the stability of the complex. Furthermore, the energy profiles of both the CD74-Bergamottin complex and the TXN-Diphenylcyclopropenone complex demonstrated that the systems gradually stabilized during the simulation, without experiencing significant fluctuations (**Figure 6G**). The energies of both complexes remained within a relatively stable range throughout the entire simulation, thereby confirming the stability of the complexes. The hydrogen bond count profiles for both the CD74-Bergamottin complex and the TXN-Diphenylcyclopropenone complex revealed that hydrogen bonds between the compound and protein gradually formed and remained stable over time (**Figure 6H**). This indicated that the interactions between them were sustained and stable throughout the molecular dynamics simulation. In addition, the binding free energy was calculated using MM/PBSA. The average binding free energy of the CD74-bergamottin complex was -152.32 kJ/mol (**Figure 6I**), and that of the TXN-diphenylcyclopropenone complex was -133.2 kJ/mol (**Figure 6J**), indicating overall binding stability during the 100 ns molecular dynamics simulations. In summary, based on computational docking and molecular dynamics simulations, bergamottin and diphenylcyclopropenone were predicted to exhibit good binding affinities for CD74 and TXN, respectively. It should be emphasized that these computational simulation results are not yet sufficient to demonstrate any functional activity or therapeutic benefits. Therefore, future experimental studies, such as binding affinity assays and cell-based functional assays, are needed to validate their interactions with target proteins and elucidate their biological effects.

**Table 3:** The binding free energy between biomarkers and drugs.

Gene	Drug	Binding free energy (kcal/mol)
CD74	Bergamottin	-6.24
TXN	Diphenylcyclopropenone	-5.736

### 3.7 Myeloid cells were identified as a potential key cell type

**Figure 7:**
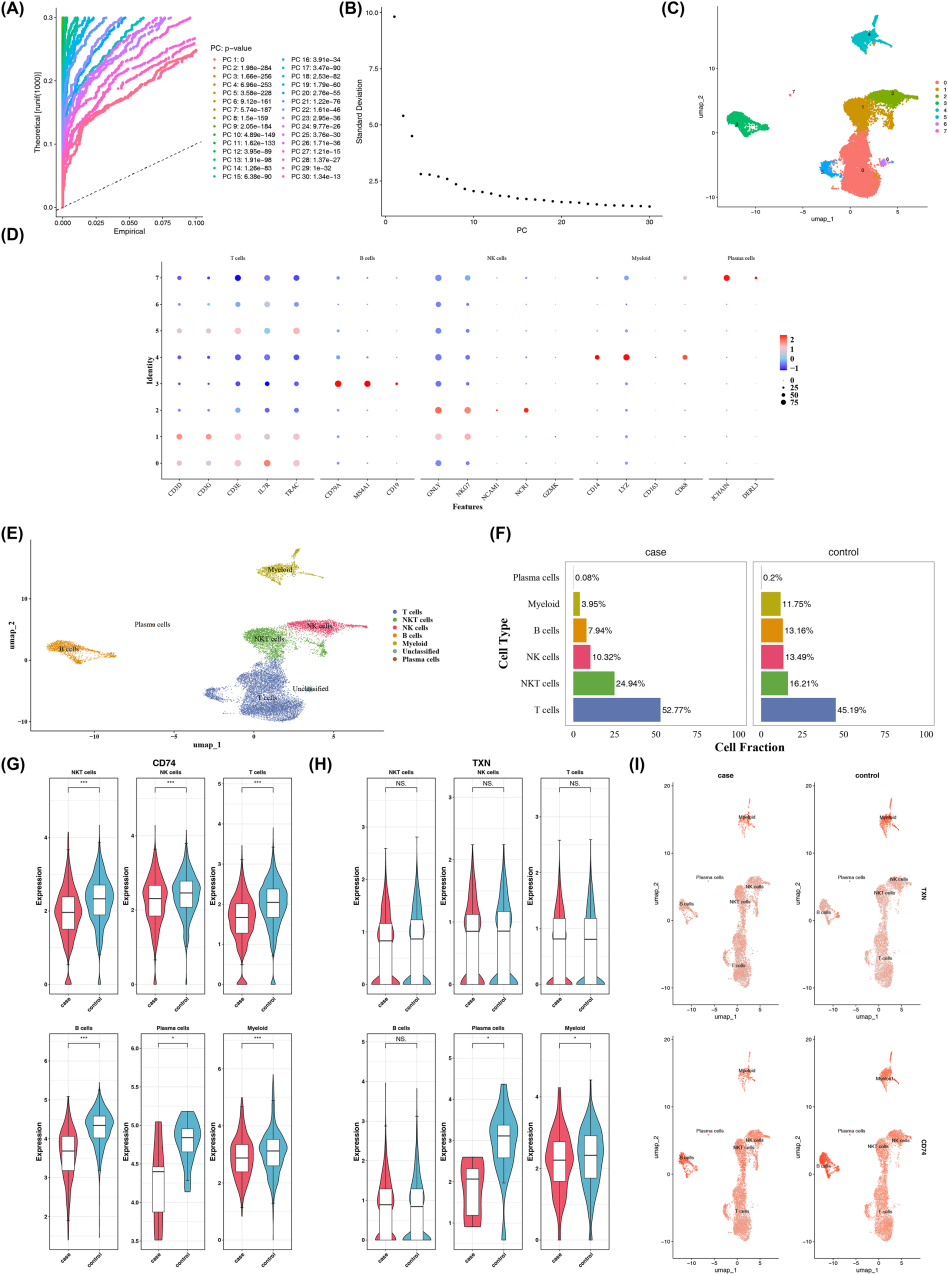
scRNA-seq data processing and annotated cell types characterization. **(A)** JackStraw plot indicating the significance of the top 30 PCs for the scRNA-seq data analysis. **(B)** Scree plot showing the standard deviation of each of the first 30 PCs. **(C)** UMAP clustering plot revealing the eight distinct cell clusters identified in the scRNA-seq dataset. **(D)** Dot plot displaying the expression of key immune cell markers across various cell types, including T cells, B cells, NK cells, myeloid cells, and plasma cells. The color scale indicates the relative expression of each marker. **(E)** UMAP clustering plot of the cell types identified by scRNA-seq, with distinct markers for T cells, B cells, NK cells, myeloid cells, and plasma cells. **(F)** Cell proportion analysis showing the distribution of T cells and NK T cells in both RA and control groups. **(G)** Violin plot illustrating the differential expression of CD74 between cell types (NKT cells, NK cells, T cells, B cells, plasma cells, and myeloid cells) in both the RA and control groups. **(H)** Violin plot displaying the differential expression of TXN in various cell types (NKT cells, NK cells, T cells, B cells, plasma cells, and myeloid cells) in the RA and control groups. **(I)** UMAP plots showing the expression of CD74, TXN, and NKT cells across RA and control groups.

During the scRNA-seq data (GSE289019) processing, an initial dataset comprising 15,849 cells and 30,054 genes underwent QC procedures (**Supplementary Figure 2A**). Following quality assessment and filtering, a refined dataset containing 14,884 cells and the same 30,054 genes was retained (**Supplementary Figure 2B**). Subsequently, the top 25 PCs were selected for subsequent analysis (**Figures 7A, B**). UMAP-based clustering resolved the cells into eight distinct clusters (**Figure 7C**). These were clearly annotated as T cells (CD3D, CD3G, CD3E, IL7R, TRAC), B cells (CD79A, MS4A1, CD19), NK cells (GNLY, NKG7, NCAM1, NCR1, GZMK), myeloid cells (CD14, LYZ, CD163, CD68), and plasma cells (JCHAIN, DERL3) (**Figures 7D, E**). Cluster 6 cells, although showing an expression pattern adjacent to that of T cells on the UMAP plot, were classified as “unclassified” and excluded from subsequent analyses due to their lack of expression of the immune cell marker CD45 and their relatively small cell number. T cells and NKT cells exhibited relatively high proportions in both the RA and control groups (**Figure 7F**). Specifically, in RA samples, T cells constituted 52.77% of cells compared to 45.19% in controls, and NKT cells accounted for 24.94% versus 16.21%, respectively. CD74 and TXN exhibited significant expression differences between plasma cells and myeloid cells in both groups (P < 0.05) (**Figures 7G, H**). PGLYRP1 expression was absent in the scRNA-seq data (GSE289019) (**Figure 7I**). Given the low plasma cell count precluding further analysis, myeloid cells were designated as the potential key cell type for subsequent investigation. To further validate these findings, we performed cross-validation using independent single-cell data from peripheral blood mononuclear cells in the CZ CELLxGENE Discover database (https://cellxgene.cziscience.com/). The results showed that, consistent with GSE289019, TXN still exhibited significant differential expression in myeloid cells (P < 0.05) (**Supplementary Figures 3A–C**). Although the differential expression of CD74 in myeloid cells did not reach statistical significance, its expression was detectable in most myeloid cells, suggesting a certain degree of heterogeneity in its expression pattern across different datasets.

### 3.8 Cellular communication and metabolism analyses for annotated cell types

**Figure 8:**
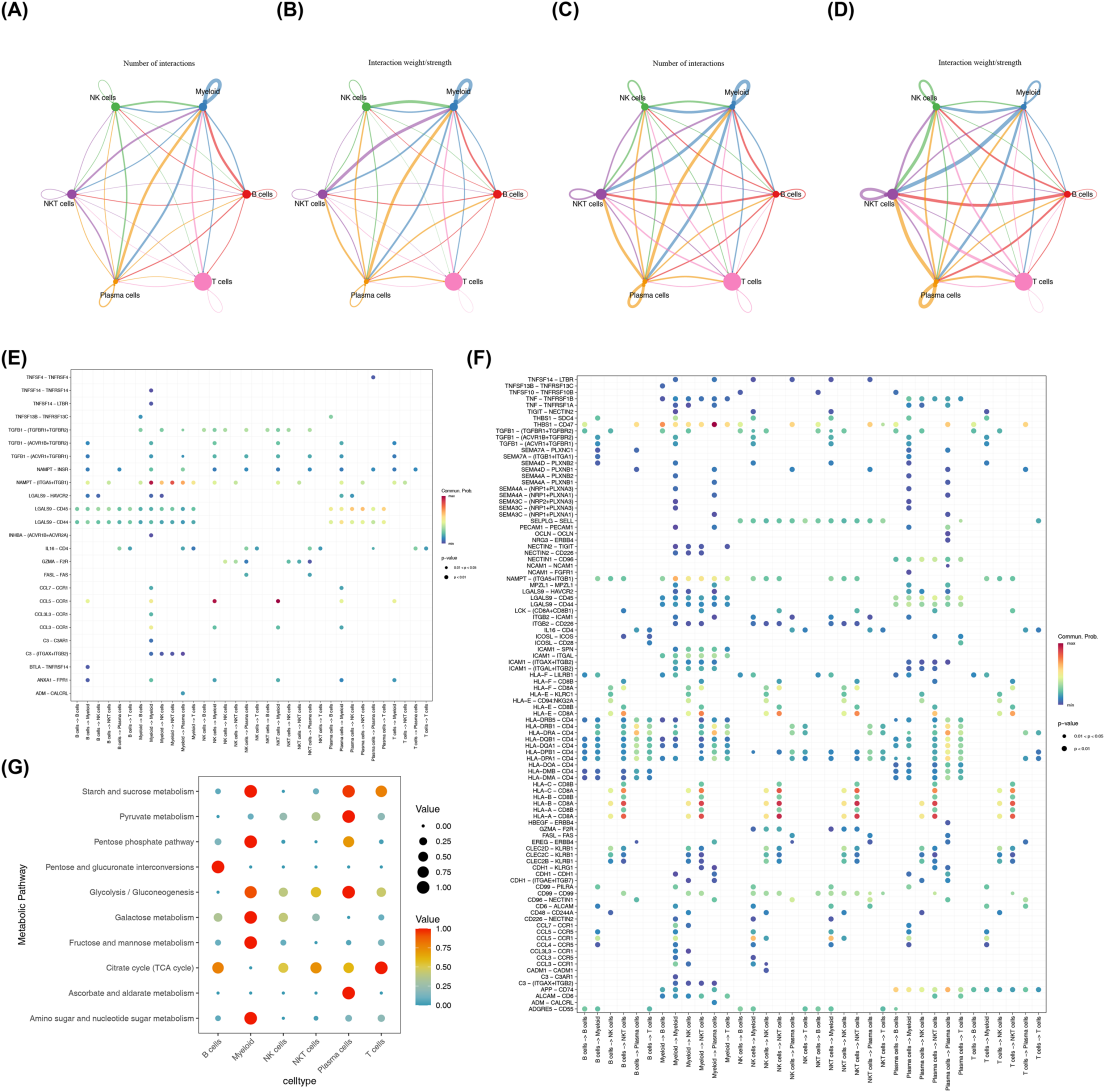
Cellular communication analysis and metabolic pathway exploration of annotated cell types. **(A)** Network diagram showing the number of interactions between immune cell types (B cells, myeloid, NK cells, NKT cells, plasma cells, and T cells) in the control group. Each line represents a connection between two cell types, and the thickness of the line correlates with the number of interactions. **(B)** Network diagram illustrating the interaction weight/strength between immune cell types in the control group, with varying colors and line thickness indicating the strength of the interactions between cells. **(C)** Network diagram displaying the number of interactions in the RA group. **(D)** Network diagram showing the interaction weight/strength in the RA group. **(E)** Bubble plot showing the ligand-receptor interactions between immune cell types in the control group. The size and color of the bubbles represent the communication probability and P value. **(F)** Bubble plot showing the ligand-receptor interactions between immune cell types in the RA group. **(G)** Metabolic pathway enrichment analysis for the annotated cell types.

Relative to the control group, the RA group exhibited significantly higher cell-cell interaction frequencies and intensities, especially among T cells, B cells, and myeloid cell populations (**Figures 8A–D**). This indicated that in the RA group, the communication activity among immune cells was notably elevated, likely closely associated with the immune dysregulation and inflammatory response characteristic of RA. Furthermore, in the control group, the CCL5-CCR1 ligand-receptor pair exhibited the highest communication probability between NK cells and myeloid cells, as well as between NKT cells and myeloid cells (**Figure 8E**). These types might have played crucial regulatory roles through mutual interactions during the immune response. The THBS1-CD47 interaction showed the strongest communication probability between myeloid cells and plasma cells in RA samples (**Figure 8F**). This observation implies a potential role for this ligand-receptor pair in perpetuating immune responses and driving inflammatory processes in the RA synovial microenvironment. Distinct metabolic profiles among cell types can correspond to their unique functional roles. Using scMetabolism, myeloid cells were found to exhibit elevated activity in pathways such as “starch and sucrose metabolism,” “pyruvate metabolism,” and “ascorbate and aldarate metabolism” (**Figure 8G**). The heightened activity in these metabolic pathways might be associated with the pivotal role of myeloid cells in immune responses, supporting their functional requirements in anti-inflammation, immune surveillance, and cellular metabolic regulation.

### 3.9 Pseudo-temporal trajectory analysis, TF activities evaluation, cell cycle analysis, and GSVA for myeloid cells

**Figure 9:**
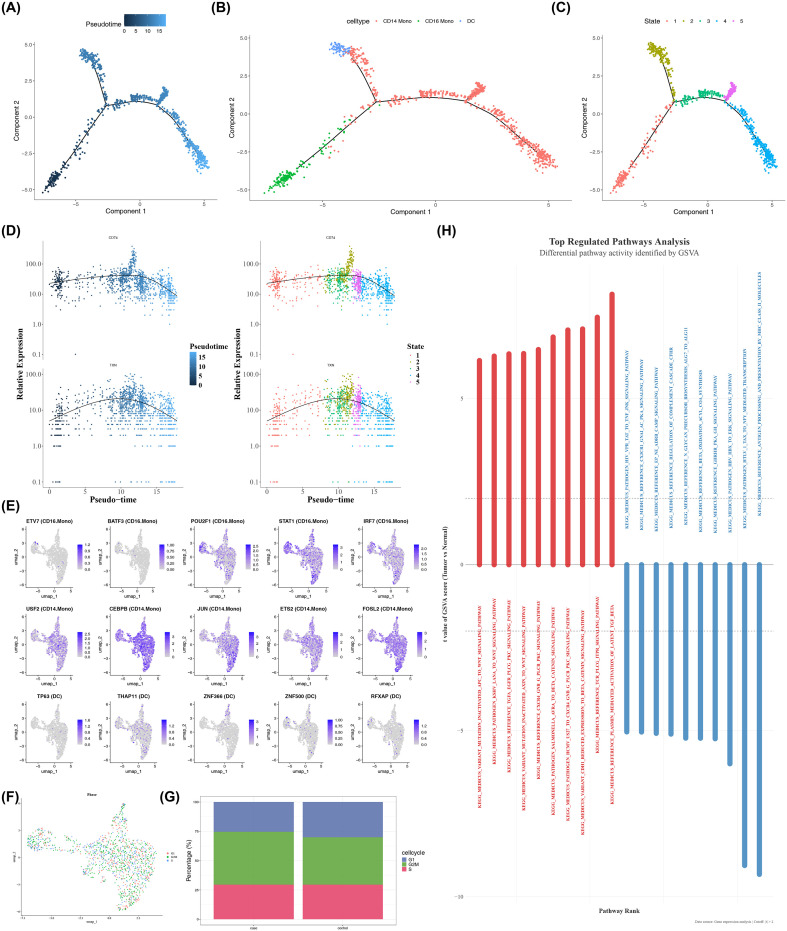
Myeloid cell differentiation, pseudo-temporal trajectory analysis, TF activities evaluation, cell cycle analysis, and GSVA. **(A)** Pseudo-temporal trajectory plot for myeloid cell differentiation, showing the progression of myeloid cells through pseudotime. The early and late stages of differentiation are represented by darker and lighter shades of blue, respectively. **(B)** Pseudotime-based analysis of myeloid cell differentiation, showing the cell differentiation stages of CD16 mono, CD14 mono, and DC populations. **(C)** State-based pseudotime trajectory analysis, depicting five distinct states in the progression of myeloid cell differentiation. **(D)** Gene expression trajectories for CD74 and TXN across myeloid cell differentiation. **(E)** TF expression patterns across myeloid cell subsets. **(F)** UMAP plot showing myeloid cells in different cell cycle phases (G1, S, and G2M). **(G)** Cell cycle phase distribution comparison between the RA and control groups. **(H)** GSVA pathway activity analysis comparing the RA and control groups.

Dimensionality reduction and clustering techniques were applied to myeloid cells, followed by the utilization of the UMAP method to identify three distinct subclusters (**Supplementary Figures 4A–C**). The myeloid cells were then categorized into three separate subpopulations: CD14 mono (characterized by the expression of VCAN, S100A8, S100A9, and CD14), CD16 mono (marked by CDKN1C, CSF1R, and TCF7L2), and DC (identified by CD1C, FCER1A, and CLEC10A) (**Supplementary Figures 4D, E**). Subsequently, a pseudo-temporal trajectory analysis was performed on the myeloid cells. **Figure 9A** illustrates the temporal trajectory of myeloid cell differentiation. As pseudotime progressed, the cell differentiation pathways gradually changed. Darker blue shades denoted early differentiation stages, whereas the lightest blue represented the terminal differentiation stage. Notably, CD16 mono cells emerged at an earlier stage of differentiation, whereas CD14 mono cells were located at the terminal stage of differentiation (**Figure 9B**). As myeloid cells underwent differentiation, they progressed through five distinct states (**Figure 9C**). At the gene expression level, as myeloid cells differentiated, the CD74 and TXN exhibited upregulated expression during the initial phase, followed by a gradual downregulation in the later stages (**Figure 9D**). These findings provide preliminary insights into the dynamic expression changes of biomarkers during myeloid cell differentiation and suggest possible functional involvement that merits further study. Further predicting the specific TFs of myeloid cell subsets, it was found that TFs such as POU2F1, STAT1, and IRF7 exhibited high expression in CD16 mono cells, whereas TFs like USF2, CEBPB, and JUN showed strong expression in CD14 mono cells (**Figure 9E**). The expression patterns of these TFs suggested differences in gene regulation among distinct immune cell subsets. Furthermore, myeloid cells exhibited a distinct distribution in the UMAP space, with cells in the G1, S, and G2M phases displaying differential spatial arrangements (**Figure 9F**), suggesting variations in their activity levels and distribution characteristics across different cell cycle stages. In the RA group, the proportion of myeloid cells in the G2M phase was significantly increased, while the G1 phase proportion was decreased, relative to the control group (**Figure 9G**). This change suggested enhanced proliferative and mitotic activity of myeloid cells in the RA group, suggesting that rapid proliferation and activation of myeloid cells may be important characteristics of the immune response in the immunological milieu of RA. Additionally, GSVA analysis revealed significant pathway differences between the RA and control groups (**Figure 9H**). Significant activation of the “TCR PLCG ITPR signaling pathway” and “CXCR4 GNB G PLCB PKC signaling pathway” was observed in RA. These findings suggest that T-cell receptor signaling and CXCR4-associated cascades may play important roles in RA pathogenesis and may be associated with aberrant immune activation, chronic inflammation, and leukocyte recruitment to inflammatory foci. At the same time, pathways including “antigen processing and presentation by MHC class II molecules” and “HTLV-1 TAX TO NFY mediated transcription” were significantly suppressed in the RA group. This likely indicated that the function of immune cells in RA patients during antigen processing and presentation was impaired, thereby affecting the immune system’s recognition of and response to exogenous antigens.

### 3.10 RT-qPCR, WB, and IHC analyses of CD74, PGLYRP1, and TXN expression

**Figure 10:**
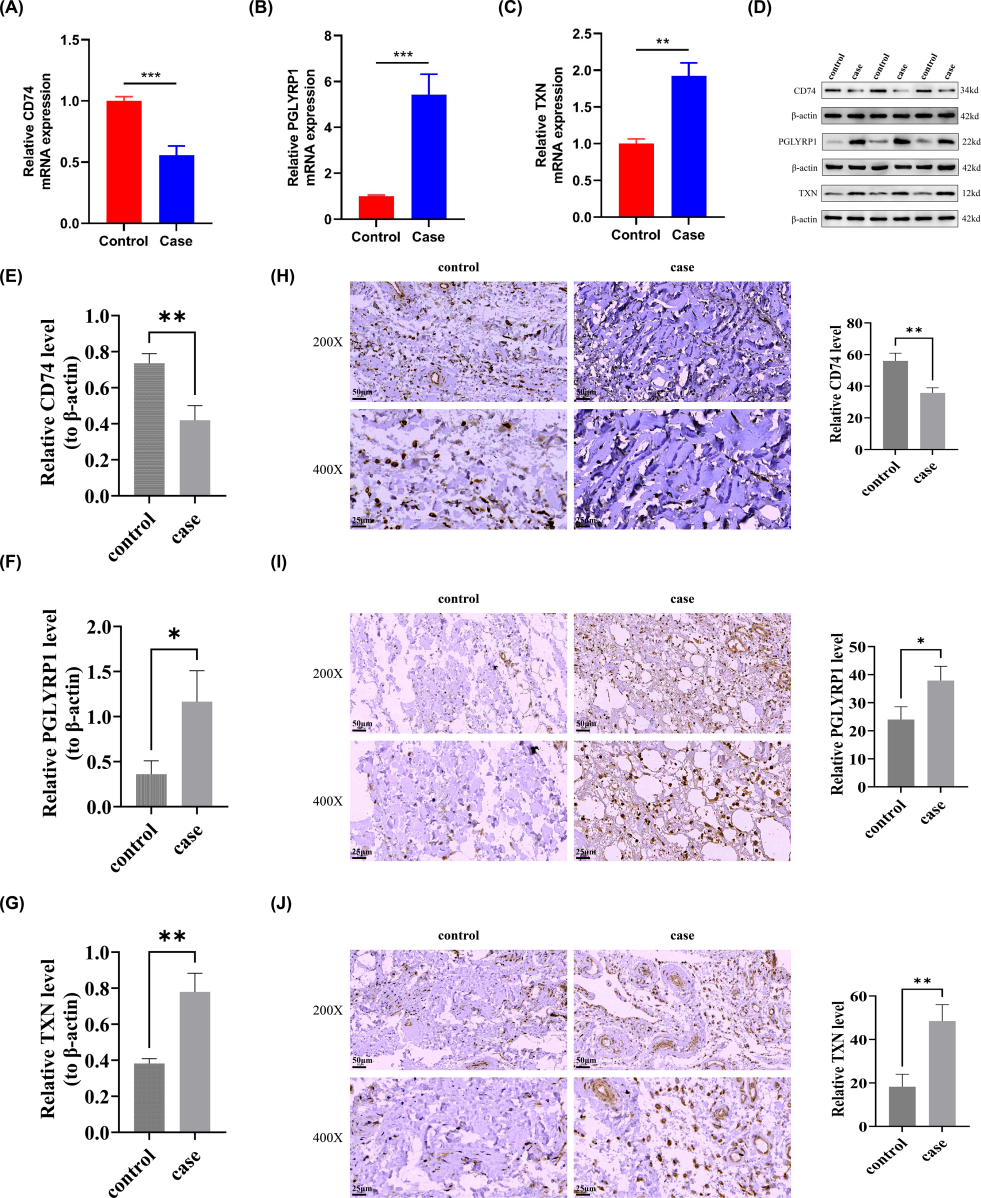
Expression of CD74, PGLYRP1 and TXN in peripheral blood PBMCs (RT-qPCR and WB) and synovial tissues (IHC). **(A–C)** mRNA expression levels of PGLYRP1, TXN, and CD74 in human peripheral blood mononuclear cells (PBMCs). Compared with the healthy control group, CD74 expression was significantly downregulated (***P < 0.001), whereas PGLYRP1 and TXN expression were markedly upregulated (**P < 0.01 and ***P < 0.001, respectively) in the RA group, with all differences being statistically significant. Each group contained 5 biological replicates, with each sample run in triplicate. Data are shown as mean ± SD. **(D)** Representative Western blot images showing the protein expression levels of CD74, PGLYRP1 and TXN in the control and case groups. β-actin was used as an internal loading control. **(E)** Quantitative analysis of CD74 protein expression normalized to β-actin in the control and case groups. **P < 0.01. Each group contained 3 biological replicates; no technical replicates were performed. Data are shown as mean ± SD. **(F)** Quantitative analysis of PGLYRP1 protein expression normalized to β-actin in the control and case groups. *P < 0.05. **(G)** Quantitative analysis of TXN protein expression normalized to β-actin in the control and case groups. **P < 0.01. **(H)** IHC staining of CD74 in synovial tissues from the control and case groups. Representative images are shown at 200× and 400× magnifications. Brown-yellow staining indicates positive expression, and nuclei were counterstained with hematoxylin. The bar graph shows the semi-quantitative analysis of CD74 expression. **P < 0.01. **(I)** IHC staining of PGLYRP1 in synovial tissues from the control and case groups. Representative images are shown at 200× and 400× magnifications, with corresponding semi-quantitative analysis. *P < 0.05. **(J)** IHC staining of TXN in synovial tissues from the control and case groups. Representative images are shown at 200× and 400× magnifications, with corresponding semi-quantitative analysis. **P < 0.01. Each group contained 3 biological replicates; for each section, three random fields were analyzed. Data are mean ± SD.

The relative mRNA expression levels of CD74, PGLYRP1, and TXN in human PBMCs are presented below. Grayscale values were quantified using ImageJ software, and statistical bar charts were generated with Prism. Compared with the control group, CD74 expression was significantly downregulated (***P < 0.001), and PGLYRP1 and TXN expression were significantly upregulated (P < 0.01, ***P < 0.001) (**Figures 10A–C**).

The WB results demonstrated that the target protein bands in all groups were clear, with molecular weights consistent with theoretical values. β-actin exhibited stable expression and was thus suitable as an internal reference for normalization analysis. Protein expression analysis revealed significant downregulation of CD74 and upregulation of PGLYRP1 and TXN in the case group relative to controls (**Figure 10D**). Band intensity quantification validated these changes (P < 0.05) (**Figures 10E–G**). The results indicate that the applied treatments effectively altered the levels of relevant regulatory and signaling proteins, offering experimental support for subsequent molecular investigations. IHC staining results revealed that PGLYRP1, TXN, and CD74 exhibited varying degrees of expression in synovial tissues (**Figures 10H–J**). The positive signals were primarily manifested as brown-yellow staining, while the cell nuclei were stained blue by hematoxylin. Relative to controls, diseased synovium displayed significantly higher PGLYRP1 and TXN expression (more positive cells, stronger staining) and significantly lower CD74 expression, as confirmed by semi-quantitative image analysis (P < 0.05). These findings imply that PGLYRP1 and TXN are involved in synovial inflammation and oxidative stress under disease conditions, while reduced CD74 may reflect compromised local immune regulatory function.

## 4 Discussion

As a chronic systemic autoimmune disease, RA manifests clinically with persistent joint inflammation, pain, and functional disability (41). Nowadays, its molecular basis and immune microenvironment continue to be actively investigated (42, 43). In this study, bioinformatic screening revealed three candidate biomarkers for RA: CD74, PGLYRP1, and TXN. These biomarkers demonstrated robust diagnostic performance (AUC > 0.7 in both cohorts) and a nomogram model achieved an AUC of 0.996, supporting strong discriminative capacity. Mechanistically, enrichment and immune-infiltration analyses associated these markers to immune, inflammatory and redox-associated processes, whereas scRNA-seq highlighted myeloid cells as a key cellular population exhibiting significant expression differences in CD74 and TXN. Furthermore, qRT-PCR, western blot, and immunohistochemical analyses consistently demonstrated that CD74 expression was significantly downregulated, while PGLYRP1 and TXN were markedly upregulated in RA clinical samples compared with controls. These results validate the reliability of our bioinformatics predictions and support the potential role of these molecules as RA biomarkers, thereby providing new clues for understanding RA pathogenesis and identifying therapeutic targeting.

The CD74 gene codes for the HLA class II gamma chain, a transmembrane glycoprotein mainly present on antigen-presenting cells (APCs) like dendritic cells, macrophages, and B cells, playing a critical role in MHC II-mediated antigen processing and presentation (44, 45). Our results demonstrated significantly reduced CD74 expression in PBMCs of individuals with RA. This finding differs from earlier studies that reported increased CD74 expression in patients with RA (46). These tissue-specific differences suggest distinct regulatory mechanisms in peripheral versus synovial compartments. This discrepancy may be attributable to differences in disease stage and treatment background across patient cohorts. Previous studies have shown that gene expression profiles in RA synovium are substantially influenced by disease activity and prior medication history (47, 48), which may contribute to inconsistencies in the direction of CD74 expression. Notably, a similar CD74 downregulation in RA patient T cells (49) supports altered antigen presentation in systemic immune responses. Further analysis demonstrated that CD74 exhibited robust diagnostic performance in two independent cohorts (GSE15573 and GSE17755). Subcellular localization analysis revealed that CD74 is primarily distributed in the Golgi apparatus, nucleus, and extracellular space, consistent with its role in antigen-processing and secretory pathways. Together, these results support the potential of CD74 as a peripheral blood biomarker for RA.

PGLYRP1, a secreted pattern recognition receptor, contributes to innate immune responses by recognizing bacterial peptidoglycan (50, 51). In this study, we observed a significant upregulation of PGLYRP1 in PBMCs from RA patients, with robust diagnostic performance across two independent validation cohorts. This finding aligns with recent reports of elevated serum PGLYRP1 levels in RA, which were positively correlated with RF and ACPA titers, suggesting a link to autoimmune responses in RA (52, 53). Our experimental results confirmed the upregulation of PGLYRP1 in RA patient samples, thereby supporting the reliability of our transcriptomic analysis. PGLYRP1 was not detected in scRNA-seq data, likely due to its secretory nature, low mRNA-protein correlation (54), and primary expression in neutrophils (50), absent from PBMC datasets. GSEA analysis indicated enrichment of PGLYRP1-associated genes in the “olfactory transduction” pathway. Although this finding may appear unrelated to RA immunopathology, emerging evidence indicates that olfactory receptors are expressed in immune cells and can regulate chemotaxis and inflammatory responses. For example, activation of olfactory receptors in macrophages promotes the secretion of MCP-1 and modulates T-cell migration and tissue retention (55). Thus, the association between PGLYRP1 and olfactory transduction may reflect a potential role in regulating immune cell migration, though the precise mechanisms warrant further investigation.

TXN encodes a small redox protein that governs the intracellular redox balance and is involved in various biological functions, such as antioxidant defense, cell proliferation, and apoptosis (56–58). In this study, TXN was found to be significantly upregulated in PBMCs from RA patients, consistent with our experimental validation. TXN upregulation in RA synovial tissue (59)and serum (60) correlates with disease activity, supporting systemic redox imbalance. GSEA analysis revealed that TXN is enriched in pathways related to “oxidative phosphorylation” and “ribosome.” Oxidative phosphorylation is the primary mitochondrial pathway for ATP production; however, under inflammatory conditions, mitochondrial dysfunction can lead to excessive generation of reactive oxygen species (ROS) (61), The upregulation of TXN, a key antioxidant molecule, likely represents a compensatory cellular response to sustained oxidative stress in the chronic inflammatory milieu of RA. This interpretation is consistent with the widespread oxidative damage and mitochondrial dysfunction observed in RA pathogenesis (62, 63). Moreover, the strong diagnostic performance of TXN across two independent cohorts, along with its correlation with disease activity (60), indicates that TXN may serve not only as a biomarker for RA but also as a potential indicator for assessing disease severity.

This opposite trend does not reflect isolated molecular alterations but likely indicates a coordinated imbalance among distinct functional modules in RA immunopathology. From a functional perspective, the three markers represent different dimensions of RA pathogenesis: downregulation of CD74 is associated with impaired antigen presentation and adaptive immune regulation; upregulation of PGLYRP1 reflects innate immune activation; and upregulation of TXN suggests redox imbalance. Notably, miRNA regulatory network analysis revealed that hsa-miR-146a-5p co-regulates both CD74 and TXN (**Figure 6A**), implying a potential post-transcriptional coordination mechanism between these molecules. Furthermore, the nomogram model incorporating all three biomarkers achieved an AUC of 0.996, significantly higher than any single marker alone, statistically supporting their complementary diagnostic value.

Eight immune cell types exhibited significant infiltration differences between RA and control groups. Crucially, CD56dim NK cells displayed the highest positive correlation with CD74 and the highest negative correlation with TXN. CD56dim NK cells, the predominant peripheral subset (64), show reduced proportions and impaired cytotoxicity in RA (65, 66), potentially compromising autoreactive cell clearance and perpetuating inflammation. TXN is a key antioxidant protein that maintains cellular redox homeostasis. Its high expression in RA may represent a stress-induced or compensatory upregulation in response to persistent oxidative stress and inflammation, and it is closely associated with oxidative damage, immune dysregulation, and joint pathology (67, 68). Under conditions of high reactive oxygen species (ROS), certain NK cell subsets are more susceptible to functional suppression or even cellular damage, a phenomenon similarly documented in other immune-related diseases (62). Therefore, the opposite correlation patterns of CD74 and TXN with CD56dim NK cells may suggest that they are associated with distinct immune or metabolic pathways. We speculate that CD74 is primarily involved in antigen presentation and adaptive immune regulation, and its positive correlation with NK cells may reflect a synergistic role in maintaining immune homeostasis. In contrast, as a redox-regulatory molecule, the negative correlation of TXN may be related to the inhibitory effect of elevated oxidative stress in the RA microenvironment on NK cell function. It should be emphasized that all the above associations were derived from in silico analysis of transcriptomic data. Future direct functional experiments, such as co-culture assays, gene knockdown, or immunophenotyping, are required to further validate the potential regulatory relationships among CD74, TXN, and CD56dim NK cells.

By integrating molecular docking and molecular dynamics simulations, bergamottin and diphenylcyclopropenone (DPCP) were predicted to bind to CD74 and TXN, respectively. The binding free energies of both complexes were below −5 kcal/mol, indicating strong binding to their respective targets under the simulation conditions. Bergamottin is a major furanocoumarin found in grapefruit and has been reported to possess notable anti-inflammatory, antioxidant, and antitumor activities (63, 69, 70), with potential as a therapeutic agent for osteoarthritis (71). Notably, we observed significant downregulation of CD74 in peripheral blood from RA patients in this study; therefore, the functional consequences of its binding to bergamottin—whether agonistic or antagonistic—and the associated biological significance need to be clarified through subsequent cellular experiments. DPCP, an immunomodulator primarily used for topical treatment, has anti-inflammatory properties (72) but is also a known contact sensitizer. Systemic application in RA may carry the risk of provoking excessive immune responses or cutaneous adverse reactions. Nonetheless, given its binding potential toward TXN, this compound may serve as a chemical probe to investigate the function of TXN in RA immune cells. Its practical utility, however, must first be validated by confirming direct binding through surface plasmon resonance or cellular thermal shift assay, followed by defining its functional direction in RA relevant cellular models.

Our pilot scRNA-seq analysis, though limited by sample size, revealed differences in CD74 and TXN expression in myeloid cells between one RA patient and one control. Myeloid cells, particularly macrophages and dendritic cells, are established key players in the chronic inflammation of RA. They drive disease progression by secreting cytokines, facilitating antigen presentation, and modulating T cell activity (73–75). Studies indicate high heterogeneity among myeloid cells in RA synovial tissue, with distinct subpopulations exhibiting significant transcriptional and functional differences that may underlie diverse clinical manifestations and treatment responses (76). In the pseudotemporal analysis of key myeloid lineages, we observed that CD74 and TXN exhibit clear stage-specific dynamic changes during cellular differentiation: their expression was markedly upregulated in the early phase of differentiation and subsequently declined in the later stages. This dynamic expression pattern suggests that these biomarkers may be primarily involved in the transition of myeloid cells from a resting state to an inflammatory effector state, as well as in subsequent functional regulation. The upregulation of CD74 during mid-stage activation likely reflects enhanced antigen-presenting capacity, promoting CD4^+^ T-cell activation, whereas its downregulation in later stages suggests a reduction in this function or the initiation of negative-feedback regulation. Its subsequent decline in later differentiation may indicate a reduction in antigen presentation or the engagement of negative feedback regulation. Concurrently, the mid-stage peak of TXN, a key regulator of redox homeostasis, more likely represents a compensatory cellular response to significantly heightened oxidative stress within the inflammatory milieu. The later decline in TXN expression may signal an exhaustion of this antioxidant regulatory capacity or entry of the cells into a terminal differentiation state. Therefore, the stage-specific expression dynamics of CD74 and TXN in differentiating myeloid cells delineate a dynamic “activation-amplification-attenuation” process within the RA immune microenvironment. This provides a novel cellular kinetic perspective for understanding how immune cell functional states evolve with RA progression.

While this integrative study identified CD74, PGLYRP1 and TXN as candidate biomarkers and highlighted the pivotal role of myeloid cells in RA, several limitations should be acknowledged. First, the conclusions are largely derived from retrospective analysis of public transcriptomic repositories. Although independent cohorts were used for validation and supporting experiments were conducted with clinical specimens, the sample sizes remain limited. Meanwhile, the extremely high AUC of 0.996 in the training set suggests a risk of overfitting, and the predictive performance may be subject to a certain degree of optimistic estimation. Therefore, future validation of the diagnostic and prognostic value of these markers in prospective, large-scale, multicenter cohort studies is necessary. Second, key mechanistic insights, especially those that involve miRNA-TF regulatory networks and candidate drug interactions, are derived from computational predictions. Functional validation in cellular or animal models is required to confirm causality and delineate the precise molecular pathways involved. Third, the scRNA-seq analysis was performed on PBMCs from a relatively small number of individuals; thus, cellular heterogeneity and functional states in synovial tissue or other RA-affected compartments may not be fully represented. Further validation in larger cohorts, integrating spatial transcriptomics and multi-omics analyses of synovial specimens, is needed to elucidate the cellular dynamics and microenvironment interactions characteristic of RA.

## 5 Conclusions

In summary, by integrating bulk and single-cell transcriptomic data, this study identifies CD74, PGLYRP1, and TXN as candidate biomarkers for RA. These molecules show statistical correlations with immune−inflammatory pathways, redox balance, and immune infiltration patterns, particularly with CD56dim NK cells and myeloid populations. A biomarker-based nomogram showed high diagnostic accuracy, and computational screening nominated bergamottin and diphenylcyclopropenone as potential ligands for CD74 and TXN, respectively. In an exploratory scRNA-seq analysis based on limited samples, myeloid cells emerged as a cell type of interest, with observed dynamic expression of CD74 and TXN during differentiation. Their altered expression was confirmed in clinical samples from RA patients. Together, these findings advance the molecular understanding of RA and highlight CD74, PGLYRP1, and TXN as promising targets for diagnosis and therapy. Future work should clarify their functional mechanisms and translational potential.

## Data Availability

The datasets presented in this study can be found in online repositories. The names of the repository/repositories and accession number(s) can be found in the article/**Supplementary Material**.

## Glossary

RA: Rheumatoid arthritis
scRNA-seq: single-cell RNA sequencing
RT-qPCR: reverse transcription quantitative PCR
WB: western blotting
IHC: immunohistochemical
NSAIDs: non-steroidal anti-inflammatory drugs
DMARDs: disease-modifying anti-rheumatic drugs
TNF: tumor necrosis factor
ACPA: anti-citrullinated peptide antibodies
anti-CCP: anti-cyclic citrullinated peptide
GEO: Gene Expression Omnibus
PBMC: peripheral blood mononuclear cell
PPA: proteome-phenome-atlas
NCBI: National Center for Biotechnology Information
DEGs: Differentially expressed genes
FC: fold change
GO: Gene Ontology
KEGG: Kyoto Encyclopedia of Genes and Genomes
MFs: molecular functions
CCs: cellular components
BPs: biological processes
PPI: protein-protein interaction
STRING: Search Tool for the Retrieval of Interacting Genes
LASSO: least absolute shrinkage and selection operator
SVM-RFE: support vector machine-recursive feature elimination
ROC: receiver operating characteristic
AUC: area under the curve
HL: Hosmer-Lemeshow
DCA: Decision curve analysis
GSEA: Gene set enrichment analysis
FDR: false discovery rate
NES: normalized enrichment score
MSigDB: Molecular Signatures Database
ssGSEA: single sample Gene Set Enrichment Analysis
miRNAs: microRNAs
TFs: transcription factors
ENCODE: Encyclopedia of DNA Elements
DSigDB: Drug Signatures Database
PDB: Protein Data Bank
MDs: molecular dynamics simulations
RMSD: root mean square deviation
RMSF: root mean square fluctuation
QC: Quality Control
CCA: Canonical Correlation Analysis
PCs: principal components
UMAP: Uniform Manifold Approximation and Projection
AUCell: Area Under the Curve calculation
GSVA: Gene Set Variation Analysis
MDSCs: myeloid-derived suppressor cells
NK: natural killer
APCs: antigen-presenting cells
ROS: reactive oxygen species
Ber: bergamottin
DPCP: diphenylcyclopropenone

## References

[1] Alivernini S Firestein GS McInnes IB . The pathogenesis of rheumatoid arthritis. Immunity. (2022) 55:2255–70. doi: 10.1016/j.immuni.2022.11.009 36516818

[2] Padyukov L . Genetics of rheumatoid arthritis. Semin Immunopathol. (2022) 44:47–62. doi: 10.1007/s00281-022-00912-0 35088123 PMC8837504

[3] Finckh A Gilbert B Hodkinson B Bae SC Thomas R Deane KD . Global epidemiology of rheumatoid arthritis. Nat Rev Rheumatol. (2022) 18:591–602. doi: 10.1038/s41584-022-00827-y 36068354

[4] Ben Mrid R Bouchmaa N Ainani H El Fatimy R Malka G Mazini L . Anti-rheumatoid drugs advancements: New insights into the molecular treatment of rheumatoid arthritis. BioMed Pharmacother. (2022) 151:113126. doi: 10.1016/j.biopha.2022.113126 35643074

[5] Ibanez-Costa A Perez-Sanchez C Patino-Trives AM Luque-Tevar M Font P Arias de la Rosa I . Splicing machinery is impaired in rheumatoid arthritis, associated with disease activity and modulated by anti-TNF therapy. Ann Rheum Dis. (2022) 81:56–67. doi: 10.1136/annrheumdis-2021-220308 34625402 PMC8762032

[6] Radu AF Bungau SG . Management of rheumatoid arthritis: An overview. Cells. (2021) 10(11):2857. doi: 10.3390/cells10112857 34831081 PMC8616326

[7] Wu CY Yang HY Luo SF Lai JH . From rheumatoid factor to anti-citrullinated protein antibodies and anti-carbamylated protein antibodies for diagnosis and prognosis prediction in patients with rheumatoid arthritis. Int J Mol Sci. (2021) 22:686. doi: 10.3390/ijms22020686 33445768 PMC7828258

[8] Koper-Lenkiewicz OM Sutkowska K Wawrusiewicz-Kurylonek N Kowalewska E Matowicka-Karna J . Proinflammatory cytokines (IL-1, -6, -8, -15, -17, -18, -23, TNF-alpha) single nucleotide polymorphisms in rheumatoid arthritis-a literature review. Int J Mol Sci. (2022) 23:2106. doi: 10.3390/ijms23042106 35216226 PMC8878005

[9] Floudas A Canavan M McGarry T Mullan R Nagpal S Veale DJ . ACPA status correlates with differential immune profile in patients with rheumatoid arthritis. Cells. (2021) 10(3):647. doi: 10.3390/cells10030647 33799480 PMC8000255

[10] Chen YC Huang CM Liu TY Wu N Chan CJ Shih PY . Effects of human leukocyte antigen DRB1 genetic polymorphism on anti-cyclic citrullinated peptide (anti-CCP) and rheumatoid factor (RF) expression in rheumatoid arthritis (RA) patients. Int J Mol Sci. (2023) 24:12036. doi: 10.3390/ijms241512036 37569411 PMC10418683

[11] Jovic D Liang X Zeng H Lin L Xu F Luo Y . Single-cell RNA sequencing technologies and applications: A brief overview. Clin Transl Med. (2022) 12:e694. doi: 10.1002/ctm2.694 35352511 PMC8964935

[12] Kim MC Gate R Lee DS Tolopko A Lu A Gordon E . Method of moments framework for differential expression analysis of single-cell RNA sequencing data. Cell. (2024) 187:6393–410. doi: 10.1016/j.cell.2024.09.044 39454576 PMC11556465

[13] Thind AS Monga I Thakur PK Kumari P Dindhoria K Krzak M . Demystifying emerging bulk RNA-seq applications: The application and utility of bioinformatic methodology. Brief Bioinform. (2021) 22:bbab259. doi: 10.1093/bib/bbab259 34329375

[14] Kuksin M Morel D Aglave M Danlos FX Marabelle A Zinovyev A . Applications of single-cell and bulk RNA sequencing in onco-immunology. Eur J Cancer. (2021) 149:193–210. doi: 10.1016/j.ejca.2021.03.005 33866228

[15] Binvignat M Miao BY Wibrand C Yang MM Rychkov D Flynn E . Single-cell RNA-seq analysis reveals cell subsets and gene signatures associated with rheumatoid arthritis disease activity. JCI Insight. (2024) 9:e178499. doi: 10.1172/jci.insight.178499 38954480 PMC11343607

[16] Ritchie ME Phipson B Wu D Hu Y Law CW Shi W . Limma powers differential expression analyses for RNA-sequencing and microarray studies. Nucleic Acids Res. (2015) 43:e47. doi: 10.1093/nar/gkv007 25605792 PMC4402510

[17] Gustavsson EK Zhang D Reynolds RH Garcia-Ruiz S Ryten M . ggtranscript: An R package for the visualization and interpretation of transcript isoforms using ggplot2. Bioinformatics. (2022) 38:3844–6. doi: 10.1093/bioinformatics/btac409 35751589 PMC9344834

[18] Liu S Bian YC Wang WL Liu TJ Zhang T Chang Y . Identification of hub genes associated with spermatogenesis by bioinformatics analysis. Sci Rep. (2023) 13:18435. doi: 10.1038/s41598-023-45620-3 37891374 PMC10611713

[19] Gao CH Yu G Cai P . ggVennDiagram: An intuitive, easy-to-use, and highly customizable R package to generate Venn diagram. Front Genet. (2021) 12:706907. doi: 10.3389/fgene.2021.706907 34557218 PMC8452859

[20] Wu T Hu E Xu S Chen M Guo P Dai Z . clusterProfiler 4.0: A universal enrichment tool for interpreting omics data. Innovation (Camb). (2021) 2:100141. doi: 10.1016/j.xinn.2021.100141 34557778 PMC8454663

[21] Gu Z Gu L Eils R Schlesner M Brors B . circlize implements and enhances circular visualization in R. Bioinformatics. (2014) 30:2811–2. doi: 10.1093/bioinformatics/btu393 24930139

[22] Friedman J Hastie T Tibshirani R . Regularization paths for generalized linear models via coordinate descent. J Stat Softw. (2010) 33:1–22. doi: 10.18637/jss.v033.i01 20808728 PMC2929880

[23] Vega-Rojas A Haro C Molina-Abril H Guil-Luna S Santos-Marcos JA Gutierrez-Mariscal FM . Gut microbiota interacts with dietary habits in screenings for early detection of colorectal cancer. Nutrients. (2024) 17:84. doi: 10.3390/nu17010084 39796518 PMC11722828

[24] Robin X Turck N Hainard A Tiberti N Lisacek F Sanchez JC . PROC: An open-source package for R and S+ to analyze and compare ROC curves. BMC Bioinf. (2011) 12:77. doi: 10.1186/1471-2105-12-77 21414208 PMC3068975

[25] Núñez E Steyerberg EW Núñez J . Regression modeling strategies. Rev Esp Cardiol. (2011) 64:501–7. doi: 10.1016/j.recesp.2011.01.019 21531065

[26] Cui L Bao J Yu C Zhang C Huang R Liu L . Development of a nomogram for predicting 90-day mortality in patients with sepsis-associated liver injury. Sci Rep. (2023) 13:3662. doi: 10.1038/s41598-023-30235-5 36871054 PMC9985651

[27] Vickers AJ Elkin EB . Decision curve analysis: A novel method for evaluating prediction models. Med Decis Making. (2006) 26:565–74. doi: 10.1177/0272989X06295361 17099194 PMC2577036

[28] Kong W Han Y Gu H Yang H Zang Y . TP53 mutation-associated immune infiltration and a novel risk score model in HNSCC. Biochem Biophys Rep. (2022) 32:101359. doi: 10.1016/j.bbrep.2022.101359 36248764 PMC9563607

[29] Hänzelmann S Castelo R Guinney J . GSVA: Gene set variation analysis for microarray and RNA-seq data. BMC Bioinf. (2013) 14:7. doi: 10.1186/1471-2105-14-7 23323831 PMC3618321

[30] Liu P Xu H Shi Y Deng L Chen X . Potential molecular mechanisms of plantain in the treatment of gout and hyperuricemia based on network pharmacology. Evid Based Complement Alternat Med. (2020) 2020:3023127. doi: 10.1155/2020/3023127 33149752 PMC7603577

[31] Schmidt T Sticht C . The simultaneous treatment of PC-3 cells with the DNA-demethylating agent decitabine and S-adenosylmethionine leads to synergistic anticancer effects. Genes (Basel). (2024) 15:1634. doi: 10.3390/genes15121634 39766901 PMC11675482

[32] Van Der Spoel D Lindahl E Hess B Groenhof G Mark AE Berendsen HJ . GROMACS: Fast, flexible, and free. J Comput Chem. (2005) 26:1701–18. doi: 10.1002/jcc.20291 16211538

[33] Satija R Farrell JA Gennert D Schier AF Regev A . Spatial reconstruction of single-cell gene expression data. Nat Biotechnol. (2015) 33:495–502. doi: 10.1038/nbt.3192 25867923 PMC4430369

[34] Xia X He C Xue Z Wang Y Qin Y Ren Z . Single cell immunoprofile of synovial fluid in rheumatoid arthritis with TNF/JAK inhibitor treatment. Nat Commun. (2025) 16:2152. doi: 10.1038/s41467-025-57361-0 40038288 PMC11880340

[35] Perik-Zavodskii R Perik-Zavodskaia O Alrhmoun S Lopatnikova J Alshevskaya A Zhukova J . Single-cell multi-omics reveals the TNF-alpha activation threshold for classical monocytes by studying healthy donors and rheumatoid arthritis patients. Front Immunol. (2025) 16:1572823. doi: 10.3389/fimmu.2025.1572823 40453083 PMC12122427

[36] Jin S Guerrero-Juarez CF Zhang L Chang I Ramos R Kuan CH . Inference and analysis of cell-cell communication using CellChat. Nat Commun. (2021) 12:1088. doi: 10.1038/s41467-021-21246-9 33597522 PMC7889871

[37] Wu Y Yang S Ma J Chen Z Song G Rao D . Spatiotemporal immune landscape of colorectal cancer liver metastasis at single-cell level. Cancer Discov. (2022) 12:134–53. doi: 10.1158/2159-8290.CD-21-0316 34417225

[38] Shao S Cao S Chen Y Zhang Z Zhaohui T . Immunological features and potential biomarkers of systemic sclerosis-associated interstitial lung disease and idiopathic pulmonary fibrosis. Clin Respir J. (2025) 19:e70072. doi: 10.1111/crj.70072 40165483 PMC11959098

[39] Trapnell C Cacchiarelli D Grimsby J Pokharel P Li S Morse M . The dynamics and regulators of cell fate decisions are revealed by pseudotemporal ordering of single cells. Nat Biotechnol. (2014) 32:381–6. doi: 10.1038/nbt.2859 24658644 PMC4122333

[40] Van de Sande B Flerin C Davie K De Waegeneer M Hulselmans G Aibar S . A scalable SCENIC workflow for single-cell gene regulatory network analysis. Nat Protoc. (2020) 15:2247–76. doi: 10.1038/s41596-020-0336-2 32561888

[41] Gravallese EM Firestein GS Koscal N Ling E Longo DL Messenger LA . What is rheumatoid arthritis? N Engl J Med. (2024) 390:e32. doi: 10.1056/NEJMp2310178 38598569

[42] Huang J Fu X Chen X Li Z Huang Y Liang C . Promising therapeutic targets for treatment of rheumatoid arthritis. Front Immunol. (2021) 12:686155. doi: 10.3389/fimmu.2021.686155 34305919 PMC8299711

[43] Ke L He Q Qu J Wang X Li K Gong X . Bone-protective effects of neutralizing angiopoietin-like protein 4 monoclonal antibody in rheumatoid arthritis. Mol Ther. (2024) 32:4497–513. doi: 10.1016/j.ymthe.2024.09.031 39367607 PMC11638830

[44] Qiu Z Khalife J Ethiraj P Jaafar C Lin AP Holder KN . IRF8-mutant B cell lymphoma evades immunity through a CD74-dependent deregulation of antigen processing and presentation in MHCII complexes. Sci Adv. (2024) 10:eadk2091. doi: 10.1126/sciadv.adk2091 38996030 PMC11244530

[45] Clanchy FIL Borghese F Bystrom J Balog A Penn H Taylor PC . Disease status in human and experimental arthritis, and response to TNF blockade, is associated with MHC class II invariant chain (CD74) isoform expression. J Autoimmun. (2022) 128:102810. doi: 10.1016/j.jaut.2022.102810 35245865

[46] Sánchez-Zuno GA Bucala R Hernández-Bello J Román-Fernández IV García-Chagollán M Nicoletti F . Canonical (CD74/CD44) and non-canonical (CXCR2, 4 and 7) MIF receptors are differentially expressed in rheumatoid arthritis patients evaluated by DAS28-ESR. J Clin Med. (2021) 11:120. doi: 10.3390/jcm11010120 35011861 PMC8745239

[47] Dennis G Holweg CT Kummerfeld SK Choy DF Setiadi AF Hackney JA . Synovial phenotypes in rheumatoid arthritis correlate with response to biologic therapeutics. Arthritis Res Ther. (2014) 16(2):R90. doi: 10.1186/ar4555 25167216 PMC4060385

[48] Triaille C Tilman G Sokolova T Loriot A Marchandise J De Montjoye S . Disease activity drives transcriptomic heterogeneity in early untreated rheumatoid synovitis. Ann Rheum Dis. (2023) 82:1538–46. doi: 10.1136/ard-2023-224068 37507201 PMC10646909

[49] Lowe K Small A Song Q Hao LY Murray-Brown W Proudman S . Transcriptomic profiling of programmed cell death 1 (PD-1) expressing T cells in early rheumatoid arthritis identifies a decreased CD4 + PD-1 + signature post-treatment. Sci Rep. (2023) 13:2847. doi: 10.1038/s41598-023-29971-5 36801909 PMC9938264

[50] Luo Q Li X Zhang L Yao F Deng Z Qing C . Serum PGLYRP-1 is a highly discriminatory biomarker for the diagnosis of rheumatoid arthritis. Mol Med Rep. (2019) 19:589–594. doi: 10.3892/mmr.2018.9632 30431075

[51] Zhang M Dai R Zhao Q Zhou L An Y Tang X . Identification of key biomarkers and immune infiltration in systemic juvenile idiopathic arthritis by integrated bioinformatic analysis. Front Mol Biosci. (2021) 8:681526. doi: 10.3389/fmolb.2021.681526 34336925 PMC8316978

[52] Sewastianik T Szydlowski M Jablonska E Bialopiotrowicz E Kiliszek P Gorniak P . FOXO1 is a TXN- and p300-dependent sensor and effector of oxidative stress in diffuse large B-cell lymphomas characterized by increased oxidative metabolism. Oncogene. (2016) 35:5989–6000. doi: 10.1038/onc.2016.126 27132507

[53] Yu L Guo Q Luo Z Wang Y Weng J Chen Y . Txn inhibitor impedes radioresistance of colorectal cancer cells with decreased Aldh1l2 expression via Txn/Nf-Kappab signaling pathway. Br J Cancer. (2022) 127:637–48. doi: 10.1038/s41416-022-01835-1 35597868 PMC9381770

[54] Reimegård J Tarbier M Danielsson M Schuster J Baskaran S Panagiotou S . A combined approach for single-cell mRNA and intracellular protein expression analysis. Commun Biol. (2021) 4:624. doi: 10.1038/s42003-021-02142-w 34035432 PMC8149646

[55] Li JJ Tay HL Plank M Essilfie AT Hansbro PM Foster PS . Activation of olfactory receptors on mouse pulmonary macrophages promotes monocyte chemotactic protein-1 production. PloS One. (2013) 8:e80148. doi: 10.1371/journal.pone.0080148 24278251 PMC3836993

[56] Zhang J Duan D Osama A Fang J . Natural molecules targeting thioredoxin system and their therapeutic potential. Antioxid Redox Signal. (2021) 34:1083–1107. doi: 10.1089/ars.2020.8213 33115246

[57] Yoshida S Katoh T Tetsuka T Uno K Matsui N Okamoto T . Involvement of thioredoxin in rheumatoid arthritis: its costimulatory roles in the TNF-alpha-induced production of IL-6 and IL-8 from cultured synovial fibroblasts. J Immunol. (1999) 163:351–8. doi: 10.4049/jimmunol.163.1.351 10384135

[58] Delcheva G Stefanova K Selimov P Stankova T . Lactoferrin and thioredoxin in rheumatoid arthritis are associated with fibrinogen but not with other acute phase proteins. Int J Mol Sci. (2025) 26:8211. doi: 10.3390/ijms26178211 40943137 PMC12427996

[59] Tsuji G Koshiba M Nakamura H Kosaka H Hatachi S Kurimoto C . Thioredoxin protects against joint destruction in a murine arthritis model. Free Radic Biol Med. (2006) 40:1721–31. doi: 10.1016/j.freeradbiomed.2006.01.006 16678011

[60] Jikimoto T Nishikubo Y Koshiba M Kanagawa S Morinobu S Morinobu A . Thioredoxin as a biomarker for oxidative stress in patients with rheumatoid arthritis. Mol Immunol. (2002) 38:765–72. doi: 10.1016/s0161-5890(01)00113-4 11841836

[61] Fock EM Parnova RG . Protective effect of mitochondria-targeted antioxidants against inflammatory response to lipopolysaccharide challenge: a review. Pharmaceutics. (2021) 13:144. doi: 10.3390/pharmaceutics13020144 33499252 PMC7910823

[62] Lu Z Tian Y Bai Z Liu J Zhang Y Qi J . Increased oxidative stress contributes to impaired peripheral CD56(dim)CD57(+) NK cells from patients with systemic lupus erythematosus. Arthritis Res Ther. (2022) 24:48. doi: 10.1186/s13075-022-02731-y 35172900 PMC8848960

[63] Adorisio S Muscari I Fierabracci A Thi Thuy T Marchetti MC Ayroldi E . Biological effects of bergamot and its potential therapeutic use as an anti-inflammatory, antioxidant, and anticancer agent. Pharm Biol. (2023) 61:639–646. doi: 10.1080/13880209.2023.2197010 37067190 PMC10114982

[64] Zimmer J . CD56(dim)CD16(dim) natural killer (NK) cells: the forgotten population. Hemasphere. (2020) 4:e348. doi: 10.1097/HS9.0000000000000348 32309785 PMC7162090

[65] Kucuksezer UC Aktas Cetin E Esen F Tahrali I Akdeniz N Gelmez MY . The role of natural killer cells in autoimmune diseases. Front Immunol. (2021) 12:622306. doi: 10.3389/fimmu.2021.622306 33717125 PMC7947192

[66] Kucuksezer UC Aktas Cetin E Esen F Tahrali I Akdeniz N Gelmez MY . The role of natural killer cells in autoimmune diseases. Front Immunol. (2021) 12:622306. doi: 10.3389/fimmu.2021.622306 33717125 PMC7947192

[67] Muri J Kopf M . The thioredoxin system: balancing redox responses in immune cells and tumors. Eur J Immunol. (2023) 53:e2249948. doi: 10.1002/eji.202249948 36285367 PMC10100330

[68] Lu T Zong M Fan S Lu Y Yu S Fan L . Thioredoxin 1 is associated with the proliferation and apoptosis of rheumatoid arthritis fibroblast-like synoviocytes. Clin Rheumatol. (2018) 37:117–25. doi: 10.1007/s10067-017-3832-1 28914370 PMC5754431

[69] Bhusal A Kim JH Kim SC Hwang EM Ryu H Ali MS . The microglial innate immune protein Pglyrp1 mediates neuroinflammation and consequent behavioral changes. Cell Rep. (2024) 43:113813. doi: 10.1016/j.celrep.2024.113813 38393947

[70] Russo C Lombardo GE Bruschetta G Rapisarda A Maugeri A Navarra M . Bergamot byproducts: a sustainable source to counteract inflammation. Nutrients. (2024) 16:259. doi: 10.3390/nu16020259 38257152 PMC10819577

[71] Shen G Zhang W Tu Q Wang J . Bergamottin (Ber) ameliorates the progression of osteoarthritis via the Sirt1/Nf-Kappab pathway. Front Pharmacol. (2024) 15:1389786. doi: 10.3389/fphar.2024.1389786 38741587 PMC11089381

[72] Inoue Y Yoshino K Kudo S Kodama N Moteki H Kimura M . Preparation, solubility, and anti-inflammatory effects of a complex of diphenylcyclopropenone/β-cyclodextrin derivatives as the treatment of alopecia areata. J Pharm Pharm Sci. (2024) 27:13230. doi: 10.3389/jpps.2024.13230 39193564 PMC11348807

[73] Rajabinejad M Salari F Gorgin Karaji A Rezaiemanesh A . The role of myeloid-derived suppressor cells in the pathogenesis of rheumatoid arthritis; anti- or pro-inflammatory cells? Artif Cells Nanomed Biotechnol. (2019) 47:4149–58. doi: 10.1080/21691401.2019.1687504 31698956

[74] Yan L Liang M Yang T Ji J Jose Kumar GS Sreena Hou X . The immunoregulatory role of myeloid-derived suppressor cells in the pathogenesis of rheumatoid arthritis. Front Immunol. (2020) 11:568362. doi: 10.3389/fimmu.2020.568362 33042149 PMC7522347

[75] Zheng Y Wei K Jiang P Zhao J Shan Y Shi Y . Macrophage polarization in rheumatoid arthritis: signaling pathways, metabolic reprogramming, and crosstalk with synovial fibroblasts. Front Immunol. (2024) 15:1394108. doi: 10.3389/fimmu.2024.1394108 38799455 PMC11116671

[76] Hu X Zhang Z Long L Gu M Chen W Pan B . Deconvolution of synovial myeloid cell subsets across pathotypes and role of Col3a1+ macrophages in rheumatoid arthritis remission. Front Immunol. (2024) 15:1307748. doi: 10.3389/fimmu.2024.1307748 38601143 PMC11005452

