# Supplementary figures and images for: Identification and experimental validation of CD74, PGLYRP1, and TXN as potential biomarkers in rheumatoid arthritis: an integrative bulk and ScRNA-seq study
Source: Front Immunol. 2026 Jun 23;17:1824952. doi: 10.3389/fimmu.2026.1824952 (PMC13337702; doi:10.3389/fimmu.2026.1824952)

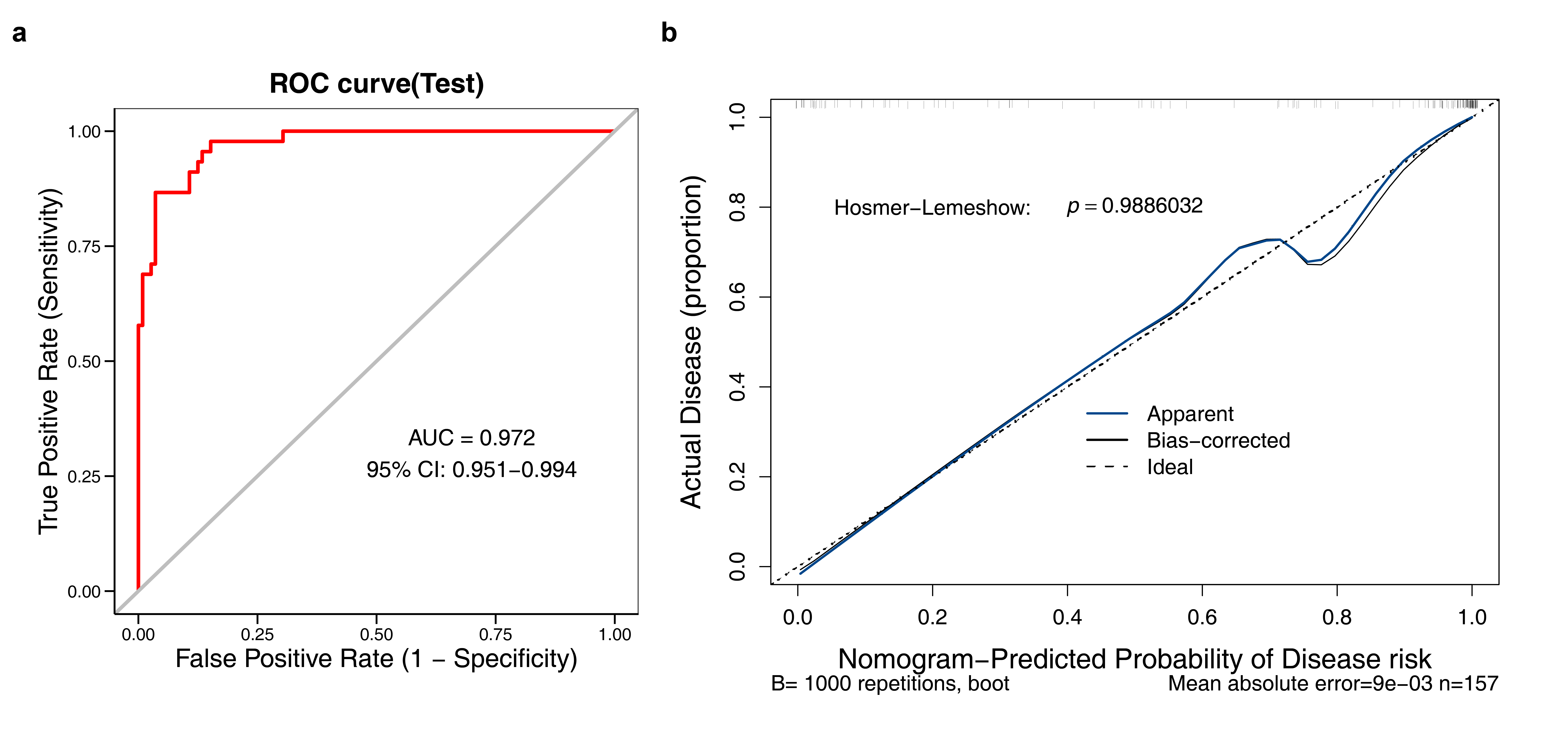

Supplement: Supplementary file 1 [file Image1.tif]

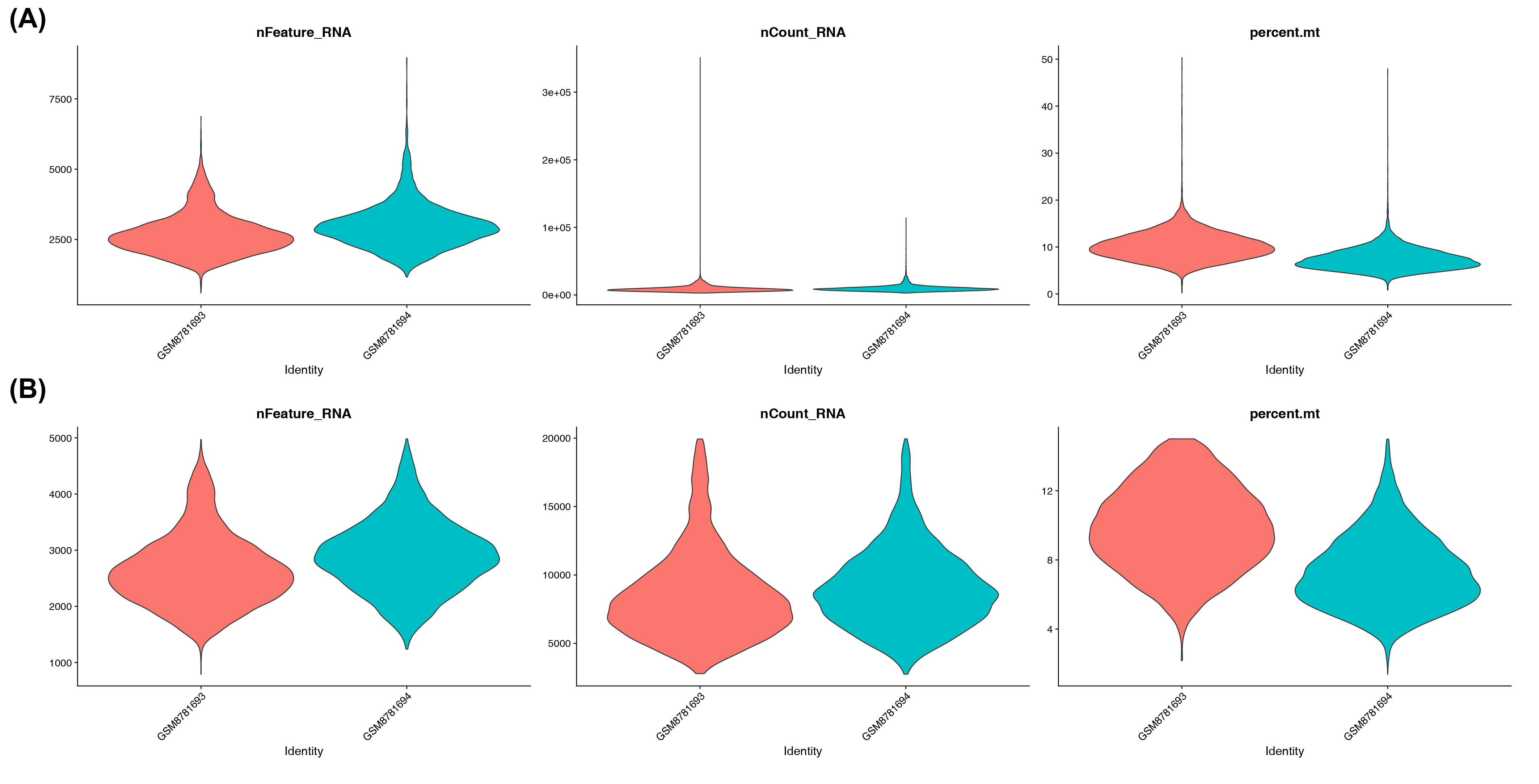

Supplement: Supplementary file 2 [file Image2.tif]

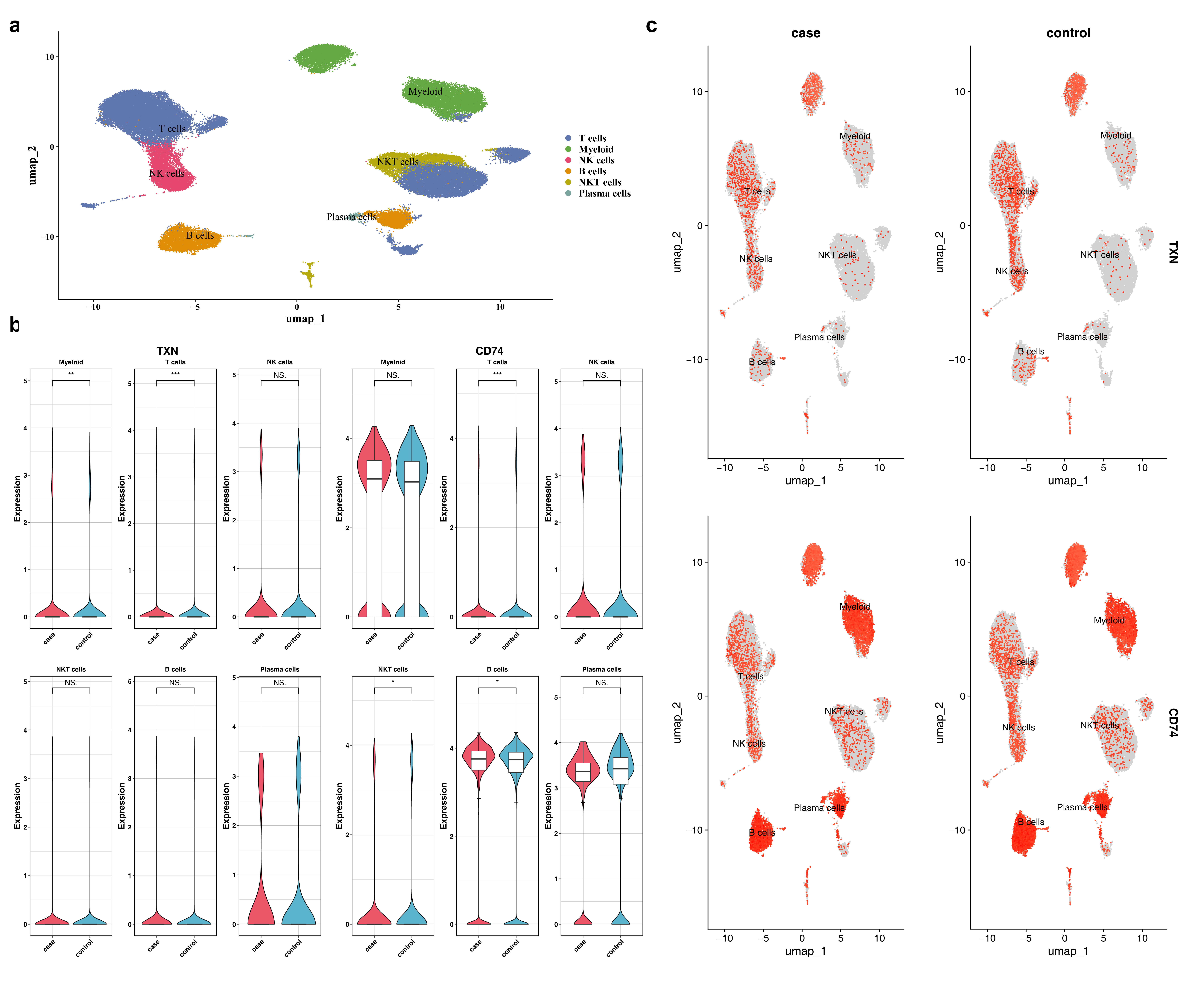

Supplement: Supplementary file 3 [file Image3.tif]

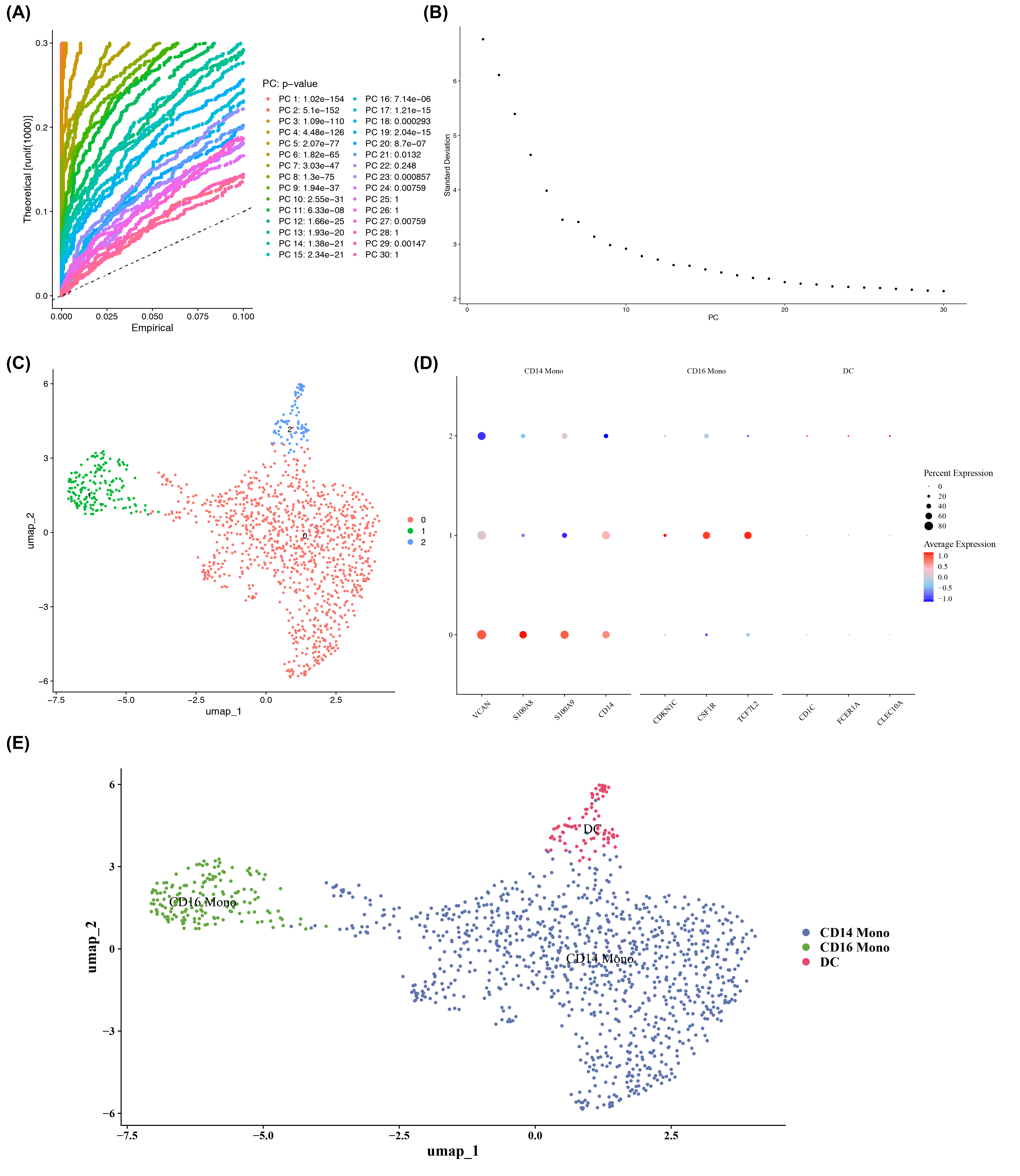

Supplement: Supplementary file 4 [file Image4.tif]
