# Supplementary material for: Identification and experimental validation of CD74, PGLYRP1, and TXN as potential biomarkers in rheumatoid arthritis: an integrative bulk and ScRNA-seq study
Source: Front Immunol. 2026 Jun 23;17:1824952. doi: 10.3389/fimmu.2026.1824952 (PMC13337702; doi:10.3389/fimmu.2026.1824952)
Supplement: Supplementary file 11 [file SupplementaryFile1.docx]

Supplementary Material

# Supplementary Figures and Tables

## Supplementary Figures

**Supplementary Figure 1.** ROC curve **(A)** and calibration curve **(B)** of the nomogram in the validation cohort GSE17755

**Supplementary Figure 2.** QC analysis of scRNA-seq data. **(A)** Violin plot showing the distribution of RNA features before QC processing. The plot presents the number of features (nFeature_RNA), total RNA count (nCount_RNA), and mitochondrial gene percentage (percent.mt) for each sample. **(B)** Violin plot illustrating the distribution of RNA features after QC processing.

**Supplementary Figure 3.** Validation of CD74 and TXN expression in myeloid cells based on the CZ CELLxGENE Discover database. **(A)** UMAP clustering plot of cell types. **(B)** Expression of biomarkers across different cell types. **(C)** Expression distribution of biomarkers in the UMAP plot (red indicates expression level).

**Supplementary Figure 4.** Myeloid cell subpopulations identification. **(A)** JackStraw plot showing the significance of the top 30 PCs for myeloid cells after dimensionality reduction. **(B)** Scree plot showing the standard deviation across the top 30 PCs. **(C)** UMAP plot of myeloid cells, revealing three distinct subclusters based on gene expression profiles. **(D)** Dot plot showing the expression of key marker genes (VCAN, S100A8, S100A9, CD14 for CD14 mono; CDKN1C, CSF1R, TCF7L2 for CD16 mono; CD1C, FCER1A, CLEC10A for DC) across the identified myeloid cell subclusters. **(E)** UMAP plot highlighting the three identified myeloid subpopulations (CD14 mono, CD16 mono, and DC) based on marker gene expression.

- 1. **Supplementary Tables**

**Supplementary Table 1.** List of 1,506 protein-coding genes associated with RA.

**Supplementary Table 2**. GO term enrichment analysis for 27 candidate genes associated with RA.

**Supplementary Table 3.** KEGG pathway enrichment analysis for 27 candidate genes in RA.

**Supplementary Table 4.** Correlation analysis between differentially infiltrated immune cell types and CD74, PGLYRP1, TXN expression.

**Supplementary Table 5.** miRNAs predicted to target CD74, PGLYRP1, and TXN in RA.

**Supplementary Table 6.** Predicted drugs targeting CD74, PGLYRP1, and TXN.
